# ﻿Annotated checklist of *Rubus* L. (Rosaceae) from South America

**DOI:** 10.3897/phytokeys.247.127527

**Published:** 2024-10-09

**Authors:** David A. Espinel Ortiz, Katya Romoleroux, Tim Böhnert, Maximilian Weigend

**Affiliations:** 1 Bonn Institute of Organismic Biodiversity, University of Bonn, Meckenheimer Allee 170, 53115 Bonn, Germany University of Bonn Bonn Germany; 2 Laboratorio de Botánica Sistemática, Herbario QCA, Facultad de Ciencias Exactas y Naturales, Pontificia Universidad Católica del Ecuador, Av. 12 de Octubre 1076 y Vicente Ramón Roca, 170525 Quito, Ecuador Pontificia Universidad Católica del Ecuador Quito Ecuador

**Keywords:** Amazonia, Andes, Rubeae, synonymy, taxonomy, typification

## Abstract

The diversity of *Rubus* in South America is much understudied and a taxonomic framework needs to be established as a basis for future revisionary and phylogenetic work. Our review identified 110 names based on South American specimens which were published since 1767. Each name was then classified according to its botanical description and type material. Additionally, where necessary, we suggest appropriate lecto-, neo-, or epitypes. A comprehensive list of synonyms is provided and representative herbarium specimens for each country are cited to tentatively document geographical range. In total, we accept 46 species of *Rubus* recorded across South America, propose 19 new synonyms, restore *R.organensis*, previously a synonym of *R.brasiliensis*, provide a replacement name for the latter, and include new country records of *R.azuayensis*, *R.laegaardii* and *R.rusbyi*. This checklist serves as an essential starting point for future monographic and evolutionary studies on *Rubus* in South America.

## ﻿Introduction

*Rubus* L. is the most diverse genus in Rosaceae, with ca 900–1500 species distributed worldwide (Focke 1910, 1911b, 1914; Christenhusz and Byng 2016; Huang et al. 2023; POWO 2024). The genus is believed to have originated in North America and then colonized Eurasia, Central and South America during the Miocene, ultimately reaching Oceania through long-distance dispersal (Carter et al. 2019). However, its species-level taxonomy is challenging due to morphological diversity, hybridization, polyploidy and apomixis (Sochor et al. 2022). Recent molecular studies have helped to identify the major clades in *Rubus* and revise the infrageneric classification of the genus (Carter et al. 2019; Huang et al. 2023), but South American *Rubus*, particularly Andean species, are underrepresented in these studies.

South American species were until recently classified in three subgenera: R.subg.Rubus, R.subg.Comaroposis (Rich. ex Nestl.) Focke, and R.subg.Orobatus (Focke) Focke (Romoleroux 1996; Carter et al. 2019; Espinel-Ortiz and Romoleroux 2021), but according to Huang et al. (2023), all the South American species fall into Rubussubg.Rubus. Overall, the taxonomy and systematics of *Rubus* in South America on a larger scale is not well documented. Most of the recent literature has focused on limited geographical areas (e.g., countries, departments, provinces or regions) when describing new species (Moreno-Medina et al. 2020; Espinel-Ortiz and Romoleroux 2021), reporting introduced or rediscovered taxa (Espinel-Ortiz and Romoleroux 2020; Espinel-Ortiz et al. 2023), and catalogs or revisions (Forzza et al. 2010; Romoleroux et al. 2014; IPNI 2024; POWO 2024; Tropicos.org 2024). Only two historical studies cover this taxon on a global scale: The first one is the multivolume monograph of *Rubus* by Focke (1910, 1911b, 1914), which was a reference for the genus diversity worldwide, but is outdated by now, not least due to the plethora of names published in the past 100 years. The second study is a recent compilation of *Rubus* taxa considered as distinct (Huang et al. 2023), which unfortunately omits recently described or rediscovered species and neither lists synonyms nor taxonomic types. Both studies represent important milestones towards a comprehensive taxonomic treatment of the genus, but are only of limited use for future work on the South American species.

There is no recent publication summarizing taxonomic diversity and nomenclature of *Rubus* in South America, especially with the recent additions, nor are many of the names effectively typified. The present study aims at a) clarifying the names of *Rubus* in South America, to identify types and tentatively assign synonyms, b) providing a clear starting point for future revisionary, monographic and phylogenetic work on the genus in South America. To this end, we compiled a critical taxonomic checklist of the genus in South America, which includes all the published names in *Rubus* based on South American specimens, their taxonomic identity and typification, as well as the species distribution ranges as currently understood.

## ﻿Material and methods

We compiled the names of South American *Rubus* mentioned in the taxonomic literature (Macbride 1930; Fuks 1984; Romoleroux 1996; Romoleroux et al. 2014; IPNI 2024; POWO 2024; Tropicos.org 2024), tracing back each original publication with the help of the Biodiversity Heritage Library (https://www.biodiversitylibrary.org/). Across sources, we reviewed all the names where South America (e.g., “*Americaaustralis*”), or countries of South America were mentioned, either to search for type material and representative specimens for the distribution assessment.

We have personally checked the collections of *Rubus* of the following South American herbaria: CPUN, CUZ, HA, HUT, HUTI, LOJA, MA, Q, QAP, QCA, QCNE, QPLS, USM, and digitized material from: A, AAU, ASU, B, BM, BR, COL, CORD, E, F, FURB, G, GH, GOET, HAL, K, L, LD, LINN, M, MBM, MICH, MO, MPU, NY, P, PH, PRC, PUL, S, SPF, TEX, U, US, W, WU, Z. All the acronyms cited here followed those registered in Index Herbariorum (Thiers 2024).

Each species in this checklist has at least one cited specimen per country in South America, and notes on the typification and synonymy are provided where required. Herbarium acronyms are followed by a barcode, when available. Furthermore, some specimens (e.g., D. Espinel-Ortiz & H.G. Abad 281) were mounted on more than one herbarium sheet, and/or have additional dry or alcohol material, each one with its own herbarium barcode. For such specimens, we provide all the herbarium barcodes for each part when available. We have only included the herbarium specimens that we were able to revise; therefore, we do not provide information on their duplicates that we were unable to examine, unless they are considered as types, original or possible original material.

All the proposed nomenclatural actions in this checklist are based on the International Code of Nomenclature for algae, fungi, and plants (Turland et al. 2018), hereafter referred to as ICN. We include designations (names not validly published) inside quotation marks in the synonymy where appropriate. Citations of types, original material, and possible original material include an exclamation mark if the material has been seen personally, [image!] when checked from digitalized material, or (n.v.) when not seen. Within the taxon citation, we cited all the syntypes if a lectotype is designated among the extant syntypes. Additionally, ≡ is used for homotypic synonyms and = for heterotypic synonyms. The specimens cited in the distribution section are organized geographically by country from North to South and West to East. In taxonomic notes, we include information about synonymy; we also added “syn. nov.” to each name treated here as a new synonym. A list of the accepted species, the compiled names/designations and the type specimens are provided as supplementary material (See Suppl. material 1).

## ﻿Checklist of the South American *Rubus* species

### ﻿1. *Rubusacanthophyllos* Focke, Abh. Naturwiss. Vereins Bremen 4:161. 1874.

= *Rubusjelskii* Fritsch in Szyszyl., Diss. Cl. Math.-Phys. Acad. Litt. Cracov. 29: 220. 1894. • Type. Peru. Cajamarca: Cutervo, May 1879, *C. de Jelski 6* (lectotype, designated by Romoleroux 1996, pg. 10: B-10-0278049 [image!]).

**Type. Venezuela. Mérida**: Sep 1846 (fl), *N. Funck & L.J. Schlim 1142* (holotype: LE-00018277 [image!]; isotype: P-00682371 [image!]).

**Nomenclature notes.** In the protologue, Focke (1874) cited a specimen at LE, which we recognize as the holotype of *R.acanthophyllos* according to Art. 9.1 of the ICN. Additionally, we found a duplicate kept at P, which we recognize as an isotype of *R.acanthophyllos*.

**Taxonomic notes.** The synonymy follows the revision of Romoleroux (1996).

**Specimens examined. Colombia. • Boyacá**: Municipio Duitama, El Carmen, vía a Virolín, 3400–3500 m, 19 Nov 1994 (fl), *J. Betancur 5634* (COL-000057912). • **Unknown**: El Boquerón bei Bogotá, páramos, 3200–3700 m, Jul 1929 (fl), *C. Troll 3774* (B-10-0248179). **Venezuela. • Trujillo**: Parque Nacional Guaramacal, 09°16.700'N, 70°08.650'W, 2700–2800 m, Jan–Feb 1996 (fl), *B. Stergios & L. Zambrano 17746* (US-00603198). **Ecuador. • Loja**: Desvío a Fierro Urcu, aprox. 9 km desde la carretera E35, 03°42.842'S, 79°18.642'W, 3449 m, 14 Nov 2019 (fl, fr), *D. Espinel-Ortiz & E. Bastidas-León 193* (QCA-244943, QCA-7010888). **Peru. • Cajamarca**: Hualgayoc, 06°45.680'S, 78°36.018'W, 3523 m, 27 May 2014 (fr), *J. Montoya, E. Linares & A. Galán 3755* (USM-298275).

**Distribution.***Rubusacanthophyllos* is recorded along the Andes of Venezuela, Colombia, southern Ecuador and Peru.

### ﻿2. *Rubusadenothallus* Focke in Herzog, Meded. Rijks-Herb. 19: 56. 1913.

**Type. Bolivia.** Río Sanjana, near Calacheca, 3500 m, Jan 1911 (fl), *T. Herzog 2399* (lectotype, designated by Romoleroux 1996, pg. 31: L-0019722 [image!], L-0019723 [image!]).

**Specimens examined. Ecuador. • Azuay**: Vía Cuenca-Loja, desvío en la carretera E35, entrada a la comunidad “Rañas”, aprox. 37 km después de Cumbe, 03°15.213'S, 79°04.132'W, 3198 m, 13 Nov 2019 (fl, fr), *D. Espinel-Ortiz & E. Bastidas-León 187* (QCA-246116, QCA-7011142). **Peru. • Cusco**: Calca, Lares, 10 km antes del pueblo del Lares y cinco km más allá, 3200–3500 m, 30 Aug 1943 (fl, fr), *C. Vargas 3585* p.p. (CUZ-3991) **. • San Martín**: Mariscal Cáceres, lado S de Río Chochos, Río Abiseo Parque Nacional, 3400 m, 07 Jun 1986 (fr), *K. Young 3717* (CPUN-4977) **. Bolivia. • La Paz**: Inquisivi, “Aguas Calientes de Calachaca”, 9 km NW of Choquetanga, 16°48.000'S, 67°19.000'W, 3400–3500 m, 09 Mar 1991 (fl), *M. Lewis 38259* (MO-1606899).

**Distribution.***Rubusadenothallus* is recorded in the Andes of southern Ecuador, Peru and Bolivia.

### ﻿3. *Rubusadenotrichos* Schltdl., Linnaea 13(2): 267. 1839.

= *Rubusroseorum* A.Berger, J. Wash. Acad. Sci. 16(6): 161. 1926. • **Type. Ecuador. [Pichincha**]: Vicinity of Quito 26 Oct–01 Nov 1918 (fl, fr), *J.N. Rose & G. Rose 23548* (holotype: US-00095484 [image!]; isotypes: GH-40522 [image!], NY-429652 [image!]).

**Type. Mexico.** Jalapa, May 1829 (fl), *C.J.W. Schiede s.n.* (lectotype, designated here: HAL-60490 [image!]).

**Nomenclature notes.** There are some problems with the typification of *R.adenotrichos*, because the holotype was cited at different herbaria. Romoleroux (1996) cited a specimen from NY, while Tropicos.org (2024) cited specimens from G. However, neither matches the date cited in the protologue. Schlechtendal (1839) described *R.adenotrichos* from a specimen collected by Schiede in Jalapa in May. No herbarium is given in the protologue, but Schlechtendal was the director of the Botanical Garden of the Martin Luther University of Halle-Wittenberg (HAL) at the time of the description. Therefore, the original material examined by Schlechtendal is housed at HAL (Braun and Wittig 2003).

We located two specimens of *R.adenotrichos* collected by Schiede at HAL. HAL-60490 has a handwritten label which matches the protologue in locality, date of collection and flower color. The other specimen at HAL, as well as those at GOET, NY and P have only a handwritten label with the locality. It is clear that Schlechtendal used more than HAL-60490 for his description of *R.adenotrichos*. Thus, according to Art. 9.3, 9.11, and 9.12 of the ICN, lectotypification is required and we designate HAL-60490 as the lectotype of this name. Further specimens from Schiede are cited here as original material of *R.adenotrichos*.

**Taxonomic notes.** The synonymy follows the revision of Romoleroux (1996).

**Original material of *Rubusadenotrichos* Schltdl.: Mexico.** Jalapa, *C.J.W. Schiede s.n.* (HAL-107628 [image!] two sheets).

**Possible original material of *Rubusadenotrichos* Schltdl.: Mexico.** Jalapa, *C.J.W. Schiede s.n.* (GOET-010090 [image!], NY-429609 [image!], P-00682372 [image!]).

**Specimens examined. Venezuela. • Trujillo**: Boconó, Parque Nacional Guarmacal, casa Vicuyal, 2100 m, 12 Apr 2003 (fl), *B. Stergios, L.J. Dorr, S.M. Niño & R. Caracas 20169* (US-00728400). **Colombia. • Putumayo**: Valle de Sibundoy, 1.5 km S Sibundoy, 2200 m, 21 Sep 1963 (fl), *M.L. Bristol 1397* (COL-00197822). **Ecuador. • Imbabura**: Cotacachi, vía a la laguna de Cuicocha, entrando por la carretera Cotacachi-Quiroga-Cuicocha, 00°17.563'N, 78°20.918'W, 3045 m, 03 Oct 2020 (fl), *D. Espinel-Ortiz, M.P. Ortiz, M.A. Espinel-Ortiz & C. Castillo 246* (QCA-246116, QCA-7011142). • **Pichincha**: Chillo Valley, Santa Rosa, 9600 ft, 26 Aug–02 Sep 1923 (fl), *H.E. Anthony & G.H.H. Tate 204* (US-03733224); Nono, Aug 1899 (fl), *A. Sodiro 408* (Q-3608). **Peru. • Huánuco**: Huánuco, San Pedro de Cani, 3088 m, 18 Jun 2017 (fl), *J.C. Tumbay 17* (USM-312932).

**Distribution.***Rubusadenotrichos* is reported from Mexico through Central America to the Central Andes, along the Andean Cordillera of Venezuela, Colombia, Ecuador and central Peru.

### ﻿4. *Rubusalutaceus* B.L.Moreno, Casierra & Albesiano, Revista Brasil. Fruticult. 42(2)-e542: 3. 2020.

**Type. Colombia. Boyacá**: Municipality of Gachantivá, El Carmen Farm, 2504 m, 31 Mar 2017, *B. Moreno 2* (holotype: COL (n.v.)).

**Notes.** This species is known only from the holotype.

**Distribution.***Rubusalutaceus* is only known from the type locality in the Cordillera Oriental of central Colombia.

### ﻿5. *Rubusazuayensis* Romol., Fl. Ecuador 56: 9. 1996.

**Type. Ecuador. Azuay**: The eastern Cordillera, 4–6 km, north of the village of Sevilla de Oro, 9000–1000 ft, 14 Aug 1945 (fl, fr), *W.H. Camp E-4693* (holotype: NY-39569 [image!]; isotypes: BM-000622366 [image!], US-00478811 [image!], US-01013533 [image!].

**Specimens examined. Ecuador. • Loja**: Desvío a Fierro Urcu, aprox. 5.2 km desde la carretera E35, 03°41.871'S, 79°18.257'W, 3204 m, 21 Feb 2017 (fr), *D. Espinel-Ortiz, E. Bastidas-León, K. Romoleroux & M. Hidalgo 101* (QCA-243563). **Peru. • Cajamarca**: Santa Cruz, Pulán, Cerro Campanario, alrededores de la Catarata La Cuda, 3100 m, 03 Jul 2004, *G. Iberico Vela, L. Dávila & A. Chávez Santa Cruz 752* (CPUN-23094).

**Distribution.***Rubusazuayensis* is known in southern Ecuador and northern Peru. We report here for the first time the presence of *R.azuayensis* in Peru.

### ﻿6. *Rubusbogotensis* Kunth, Nov. Gen. Sp. [H.B.K.] 6[Quarto]: 220. 1823.

≡ “Rubusbogotensisvar.normalis Kuntze”, Revis. Gen. Pl. 3[3]: 78. 1898, nom. inval.

= *Rubusadenomallus* Sodiro ex Focke, Biblioth. Bot. 18, Heft 83: 52. 1914, syn. nov. • Type. Ecuador. [Pichincha]: “Crescit prope Nono” [grows near Nono], 1887 (fr), *A. Sodiro 408* (lectotype, designated here: Q-3605!).

**Type. Colombia.** Santa Fé de Bogotá, “1370 hex”, Sep (fr), *A. von Humboldt & M.A. Bonpland s.n.* (lectotype, designated by Romoleroux 1996, pg. 36: P-00679380 [image!]).

**Nomenclature notes.**Romoleroux (1996) cited the lectotype of *R.bogotensis* in P, but we found two separate sheets at P that agreed with this typification. We cite here the barcode of the lectotype selected by Romoleroux, as only one specimen was used for the typification. The lectotype has a label from the “Herbier Humboldt & Bonpland” and a separate, handwritten label from Kunth with the species identification and locality. As it lacks a collection number, the second specimen at P without such labels is treated here as original material. Focke (1914) described *R.adenomallus* based on *Sodiro 408*, which was annotated “*Rubusfulliginosus* Sodiro” by the collector. We were unable to locate this specimen at B, but we found a collection at Q that matches the original description. Moreover, the specimen at Q has a previous handwritten annotation as “*Rubusfulliginosus* Sodiro”, the same that is mentioned in the protologue. Therefore, according to Art. 9.3, 9.11 and 9.12 of the ICN, we designate it as the lectotype of *R.adenomallus*. Kuntze (1898) used “R.bogotensisvar.normalis” when referring to the typical form of *R.bogotensis* automatically including its type. According to Art. 24.3 of the ICN, “R.bogotensisvar.normalis” is an invalid name because its final epithet is different from the corresponding higher-ranked taxon.

**Taxonomic notes.***Rubusadenomallus* is considered to be a new synonym of *R.bogotensis* because its type collection has the same trifoliolate leaves, glandular trichomes and fruits (few and big drupelets) as *R.bogotensis*.

**Original material of *Rubusbogotensis* Kunth: Colombia.** Santa Fé de Bogotá, *A. von Humboldt & M.A. Bonpland s.n.* (P-00162113 [image!]).

**Specimens examined. Venezuela. • Trujillo**: Trujillo, alrededores de Guirigay, 3200 m, Aug 1958 (fl), *L. Aristeguieta 3540* (US-00727722). **Colombia. • Cundimarca**: Cordillera Oriental, páramo de Guasca, 2800–3300 m, 17 Feb 1951 (fl), *H. García-Barriga & R.E. Schultes 13504* (COL-000197256, US-03733321). **Ecuador. • Loja**: San Lucas, desvío a Lomas de Oro, aprox. 5 km desde la carretera E35, 03°40.938'S, 79°14.897'W, 3212 m, 14 Nov 2019 (fl, fr), *D. Espinel-Ortiz & E. Bastidas-León 191* (QCA-246056, QCA-7011098 to QCA-7011100). **Peru. • Junín**: Tarma, Huasahuasi, carretera Cachiyazu-Cascas, 11°10.000'S, 75°35.617'W, 3852 m, 20 May 2021 (fl), *R. Vásquez, R. Rojas & E. Pinche 45877* (USM-330175). **Bolivia. • La Paz**: Inquisivi, “Jucumarini Trail”, between Chichipata and Jucumarini, 16°58.000'S, 67°13.000'W, 3300–3400 m, 23 Feb 1990 (fr), *M. Lewis 37125* (US-03733297).

**Distribution.***Rubusbogotensis* is recorded along the Andes of Venezuela, Colombia, Ecuador, Peru and Bolivia.

### ﻿7. *Rubusboliviensis* Focke, Abh. Naturwiss. Vereins Bremen 4: 158. 1874.

= *Rubuschagalensis* Hieron., Bot. Jahrb. Syst. 20(3, Beibl. 49): 28. 1895. • Type. Ecuador. Azuay: Chagal, Western Andes of Cuenca, 2000–2600 m, Oct (fl), *F.C. Lehman 4969* (lectotype, designated by Romoleroux 1996, pg. 46: F-V0068374F [image!]; isolectotype: K-000424913 [image!]).

= *Rubusherzogii* Focke in Herzog, Meded. Rijks-Herb. 19: 56. 1913, syn. nov. ≡ Rubusbriareussubsp.herzogii (Focke) Focke, Biblioth. Bot. 18, Heft 83: 56. 1914. • Type. Bolivia. Saimapata, 2000 m, Mar 1911 (fl), *T. Herzog 1663* (lectotype, designated here: L-0019779 [image!]).

**Type. Bolivia. [La-Paz**]: Larecaja “viciniis Soratam, ad rivum Challasuyo” [near Sorata, at the river Challasuyo], 2600 m, Aug 1857 (fl, fr), *G. Mandon 676* (lectotype, designated by Romoleroux 1996, pg. 46: W-65297 [image!]; isoloectotype: K-000424914 [image!]).

**Nomenclature notes.**Romoleroux (1996) cited the holotype of *R.chagalensis* in F, but we found another sheet from this collection in K. While Hieronymus (1895) cited *Lehman 4969* in the protologue, he omitted the herbarium that held this specimen. According to Art. 9.3 of the ICN, as no clear holotype was selected and two sheets in different herbaria are extant, lectotypification is required. In this respect, Romoleroux (1996) effectively typified the name *R.chagalensis* at that time (Art. 7.11). We correct the type status to lectotype and add the corresponding barcode. Focke (Herzog 1913) described *R.herzogii* based on two specimens: *Herzog 1663* and *Buchtien s.n.* We located *Herzog 1663* in L, and according to Arts. 9.3, 9.11 and 9.12, we designate it as the lectotype of this name. We did not locate *Buchtien s.n.*, but instead we found *Buchtien 6176*, which has the same locality information as given in the protologue. We treat this specimen as possible original material of *R.herzogii*, because we lack evidence that Focke had access to it at the time of the description.

**Taxonomic notes.** We follow the revision of Romoleroux (1996), who recognized *R.chagalensis* as a synonym of *R.boliviensis*. Furthermore, we consider *R.herzogii* as a new synonym of *R.boliviensis* based on the same indumentum, especially in branches and leaves; as well as the stipules and fruits seen in the type collections.

**Possible original material of *Rubusherzogii* Focke: Bolivia.** Cotaña, 2500 m, Nov 1911 (fr), *O. Buchtien 6176* (US-00641886 [image!]).

**Specimens examined. Ecuador. • Loja**: 12–20 km S de Yangana, 04°26.016'S, 79°08.933'W, 2320–2780 m, 14 Apr 1992 (fl), *K. Romoleroux & J.L. Luteyn 1362* (QCA-91807). **Peru. • Puno**: Sandía, entre Quinsa Cruz y Muruncunca, 1800 m, 05 Aug 1965 (fr), *C. Vargas 16361* (CUZ-5599). **Bolivia. • La Paz**: Sud Yungas, Hacienda “La Florida”, 26 May 1920 (fr), *E.W.D. Holway & M.M. Holway 654* (US-00641862).

**Distribution.***Rubusboliviensis* is recorded in the Andes of Ecuador, Peru and Bolivia.

### ﻿8. *Rubusbozae* Vargas, Revista Univ. (Cuzco) 32(84): 261. 1943.

**Type. Peru. Cusco**: Calca, Lares, inmediaciones de Pampa Corral, 3600 m, 09 Feb 1943 (fl, fr), *C. Vargas 3212* (holotype: CUZ-3981!).

**Notes.***Rubusbozae* is known only from the type collection.

**Distribution.***Rubusbozae* is recorded in southern Peru.

### ﻿9. *Rubusbriareus* Focke, Repert. Spec. Nov. Regni Veg. 9: 235. 1911.

**Type. Bolivia. La Paz**: Nor Yungas, Unduavi, 3200 m, 12 Feb 1907 (fl, fr), *O. Buchtien 640* (lectotype, designated here: US-00097866 [image!]; isolectotype: NY-429632 [image!]).

**Nomenclature notes.** In the protologue, Focke (1911a) cited the gathering *Buchtien 640*, for which we found two specimens, one at NY and the other at US. The voucher at US has flowers and fruits, therefore we designate it as the lectotype of *R.briareus* (Art. 9.3, 9.11 and 9.12 of the ICN).

**Additional specimens examined. Bolivia. • La Paz**: Yungas, 1890 (fl), *M. Bang 684* (US-00641878); Sud Yungas, San Felipe, 19 May 1930 (fl), *E.W.D. Holway & M.M. Holway 617* (US-00641864). • **Cochabamba**: Cerros de Incachaca, 2000–3000 m, 04 Oct 1922 (fl), *J. Steinbach 6068* (US-03733821).

**Distribution.***Rubusbriareus* is recorded in central Bolivia.

### ﻿10. *Rubusbullatus* Rusby, Bull. New York Bot. Gard. 4(14): 351. 1907.

≡ *Rubusbetonicifolius* Focke, Biblioth. Bot. 72: 33. 1910, nom. illeg. superfl.

**Type. Bolivia.***M. Bang 2235* (lectotype, designated here: NY-429638 [image!]); isolectotypes: E-00010689 [image!], E-00296708 [image!], F-V0068373F [image!], GH-26798 [image!], GH- 26799 [image!], K-000424877 [image!], M-0214195 [image!], MICH-111130 [image!], MO-255141 [image!], NY-429635 [image!], NY-429636 [image!], NY-429637 [image!], PH-21481 [image!], PH-21482 [image!], PUL-380 [image!], US-00097868 [image!], US-00641884 [image!], TEX-371097 [image!]), WU-27792 [image!].

**Nomenclature notes.**Rusby (1907) and Focke (1910) described *R.bullatus* and *R.betonicifolius*, respectively, based on *Bang 2235* from different herbaria. Rusby worked at NY at the time of the description, so we designate the specimen NY-429638 as the lectotype (Art. 9.1, 9.3, 9.11 and 9.12 of the ICN) of *R.bullatus*. On the other hand, Focke (1910) described *R.betonicifolius* with two gatherings: *Bang 2235* and *Weberbauer 670*. The name *R.betonicifolius* was superfluous and, therefore, illegitimate when it was published as Focke included the only gathering cited by Rusby.

**Specimens examined. Peru. • Cusco**: Urubamba, Machu Picchu, Alccamayo, 13°09.033'S, 72°30.467'W, 2500–2840 m, 24 Feb 2003 (fl, fr), *L. Valenzuela, E. Succlli & I Huamantupa 1511* (CUZ-42697). • **Unknown**: Sandía, 06 Apr 1902 (fl, fr), *Weberbauer 670* (B-10-1172571).

**Distribution.***Rubusbullatus* is recorded in southern Peru and northern Bolivia.

### ﻿11. *Rubuschloropetalus* Vargas, Revista Univ. (Cuzco) 32(84): 262. 1943.

**Type. Peru. Cusco**: Calca, entre Quishuaraní y Lares, 3200–3500 m, 30 Aug 1943, *C. Vargas 3585* (holotype: CUZ-3991! p.p.; isotypes: CUZ!, USM-13834!).

**Nomenclature notes.**Vargas (1943) cited *Vargas 3583* as the type of *R.chloropetalus*, but this collection number is apparently a typographical error: *Vargas 3585* has the exact information (locality, altitude and date) given in the protologue, corresponds to the original description and is annotated as “typus”. Therefore, according to Art. 9.2 of the ICN, we correct the collection number of the holotype. However, the holotype collection of *R.chloropetalus* has two different species mounted on the same sheet. The one referring to *R.chloropetalus* occupies most of the space and has broad leaves, while a small collection with smaller leaves, at the lower left of the sheet, is *R.adenothallus*.

**Distribution.***Rubuschloropetalus* is recorded in southern Peru.

### ﻿12. *Rubuschoachiensis* A.Berger, J. Wash. Acad. Sci. 16(6): 160. 1926.

= *Rubusgachetensis* A.Berger, J. Wash. Acad. Sci. 16(6): 160. 1926, syn. nov. • Type. Colombia. [Cundinamarca]: Camino de Gachetá, in forests, 2300 m, Jan 1920 (fl), *Brother Ariste-Joseph A543* (holotype: US-00097911 [image!]).

**Type. Colombia. Cundinamarca**: Páramo de Choachí, near Bogotá, 3700 m, 8 Aug 1922 (fl), *E.P. Killip & Brother Ariste-Joseph 11967* (holotype: US-00097876 [image!]).

**Taxonomic notes.**Berger (1926) described *R.choachiensis* and *R.gachetensis* based on two specimens from Cundimarca. We recognized both names as belonging to the same species, since their holotypes have the same indument, stipules, leaves and flowers. They differ only by a thicker branch in *R.choachiensis*, which was probably collected from a more developed individual.

**Specimens examined. Colombia. • Cundinamarca**: Subachoque, páramo de El Tablazo, 3300 m, 13 Oct 2003 (fl, fr), *M. Hernández Schmidt, J.L. Fernández-Alonso, M.C. Vélez & C.A. Agudelo 1376* (COL-000048593).

**Distribution.***Rubuschoachiensis* is recorded in the Eastern Cordillera of central Colombia.

### ﻿13. *Rubuscompactus* Benth., Pl. Hartw. [Bentham]: 129. 1844.

**Type. Ecuador. [Loja**]: “In montibus Saraguro” [mountain of Saraguro], *T. Hartweg 731* (lectotype, designated by Romoleroux 1996, pg. 24: K-000424927 [image!]; isolectotype: LD-1246155 [image!]).

**Nomenclature notes.** Hartweg’s original collections ended up in different herbaria, so we correct here the typification of the South American names of *Rubus* published in Flora Hartwegiana (Bentham 1839). We located *Hartweg 731* at K and LD, both corresponding to the information given in the protologue. Considering that Romoleroux (1996) cited the holotype of *R.compactus* at K, according to Art. 7.11 of the ICN, this was an effective typification at that time. Therefore, we here correct the type status to lectotype.

**Specimens examined. Ecuador. • Azuay**: Nabón, páramo de Chunazana, a 5.4 km desde carretera E35, 03°13.372'S, 79°07.050'W, 3170 m, 17 Aug 2023 (fl, fr), *D. Espinel-Ortiz & C. Restrepo 439* (QCA). **Peru. • Cusco**: Paucartambo, Cordillera de las tres Cruces, 3600 m, 10–11 Oct 1943 (fl), *C. Vargas 3632* (CUZ-5523).

**Distribution.***Rubuscompactus* is recorded in southern Ecuador and Peru.

### ﻿14. *Rubusconchyliatus* Focke, Meded. Rijks-Herb. 19: 54. 1913.

≡ Rubuslechlerivar.conchyliatus (Focke) Focke, Biblioth. Bot. 18, Heft 83: 21. 1914.

**Type. Bolivia.** Río Sanjana, 3400 m, *T. Herzog 2206a* (lectotype, designated here: L-0019750 [image!]).

**Nomenclature notes.** In the protologue, Focke (Herzog 1913) cited *Herzog 2206a* and omitted the specific herbarium. According to Art. 9.3, 9.11 and 9.12 of the ICN, we designate the specimen at L as the lectotype of *R.conchyliatus*.

**Specimens examined. Bolivia. • La Paz**: Sud Yungas, 19.8 km E of pass between Mururata and Illimani, 16°34.000'S, 67°45.000'W, 3500 m, 11 Dec 1983 (fl), *J.C. Salomon 11368* (U-1558220).

**Distribution.***Rubusconchyliatus* is recorded in central Bolivia.

### ﻿15. *Rubuscoriaceus* Poir., Encycl. [J. Lamarck & al.] 6(1): 237. 1804.

≡ Rubusroseusvar.coriaceus (Poir.) Ser., Prodr. [A. P. de Candolle] 2: 562. 1825.

= *Rubusstuebelii* Hieron., Bot. Jahrb. Syst. 21(3): 311. 1895. • Type. Colombia. Excursión de Popayán al páramo de Huila, Mar 1869 (fl), *A. Stübel 282f* (lectotype, designated by Romoleroux 1996, pg. 12: B-10-0248190 [image!]). • Syntype. Colombia. Excursión al Volcán de Chiles, 4000–4500 m, Jan–Feb 1870 (fl), *A. Stübel 461c* (B-10-0248190 [image!]).

= “Rubusglabratusvar.heterophyllos Sodiro ex Benoist”, Bull. Soc. Bot. France 90: 15. 1943 (as “*heterophyllus*”), nom. nud., syn. nov.

**Type. Peru.***J. Dombey s.n.* (holotype: P-00678397 [image!]).

**Nomenclature notes.**Poiret (1804) cited all his new names of South American species of *Rubus* at the Jussieu Herbarium. We have located the holotype and isotype of *R.coriaceus* at P, but only the holotype has a label from the “Herbier D’Antoine Laurent de Jussieu”. According to Art. 9.1 of the ICN, we accept this specimen as the holotype of *R.coriaceus*, but as it has no collection number, we cannot confirm any duplicates. Therefore, the other specimen at P is treated here as possible original material. Benoist (1943) cited *Rivet 314* as “R.glabratusvar.heterophyllos”, but omitted the description and diagnosis. Thus, “R.glabratusvar.heterophyllos” was not validly published (Art. 38.1).

**Taxonomic notes.** The synonymy follows the revision of Romoleroux (1996), but we also recognize “R.glabratusvar.heterophyllos” as a synonym of *R.coriaceus*. It is a designation whose cited specimen we identified as *R.coriaceus*.

**Possible original material of *Rubuscoriaceus* Poir.: Peru.***J. Dombey s.n.* (P-00162122 [image!]).

**Specimens examined. Colombia. • Caldas**: Manizales, La Esperanza, Reserva Torre Cuatro,05°03.867'N, 75°23.333'W, 3500–3750 m, 22 Feb 2000 (fl), *M. Alvear, D. García & D. Alvear 733* (COL-000197376). **Ecuador. • Napo**: Límites de la Reserva Ecológica Cayambe-Coca, 00°20.000'S, 78°14.000'W, 3900 m, 18 Nov 1990 (fl, fr), *A.P. Yánez 73* (QCA-91900). • **Unknown**: Aug 1901 (fl), *A. Sodiro 407* (QPLS-6755); El Pelado, Jan 1903 (fl), *M. Rivet 314* (P-03341383). **Peru. • Lambayeque**: Ferreñafe, Incahuasi, Sinchichual, above Tungula, 06°11.248'S, 79°18.618'W, 3368 m, 24 Nov 2014 (fl, fr), *M. Weigend, J. Chacón, E.F. Rodríguez, T. Henning, L.F. García, S.N. Miranda & D.F. Paredes 9690* (USM-286340).

**Distribution.***Rubuscoriaceus* is recorded along the Andes of Colombia, Ecuador, Peru and Bolivia (Romoleroux et al. 2014).

### ﻿16. *Rubuserythroclados* Mart. ex Hook.f., Fl. Bras. (Martius) 14(2): 62. 1867.

**Type. Brazil. Minas Gerais**: “Habitat in sepibus prope Capaó ubi Tapazii inveniuntur” [habitat in the hedges near Capaó where the Tapazii are found], Feb (fl, fr), *C.F.P. von Martius 853* (lectotype, designated by Fuks 1984, pg. 13: M-0214178 [image!]). • **Syntypes. Brazil. Unknown**: “near Hamhó”, Aug 1840 (fr), *G. Gardner 4546* (syntype: K-000424880 [image!]); Villa ricca, 1829 (fr), *J.P.E. Pohl s.n.* (syntype: M-0214179 [image!]).

**Nomenclature notes.** In the protologue, Hooker (1867) cited three specimens: *Martius*, *Pohl s.n.* and *Gardner 4546*. There are two lectotypifications of the name. Fuks (1984) stated that *Martius 853* (M) is the holotype of *R.erythroclados*, and according to Art. 7.11 of the ICN, this was an effective typification at that time. The other typification comes from Canero et al. (2016), who designated the same specimen as Fuks, as the lectotype of the name. Both authors referred to the same specimen, but the correct typification is that of Fuks. The other specimens are treated here as syntypes.

**Specimens examined. Brazil. • Caldas**: Manizales, La Esperanza, Reserva Torre Cuatro, 800 m, 14 Dec 1995 (fr), *R. Wasum 11570* (US-01351292). **Argentina. • Misiones**: Gral. M. Belgrano, entre San Antonio y Bernardo de Yrigoyen, 30 Oct 1960 (fr), *L. Ariza Espinar 1180* (CORD-00098599). **Paraguay. • Unknown**: Curuguaty, Sep 1898–1899 (fl, fr), *E. Hassler 4597* (G-00640003 two sheets).

**Distribution.***Rubuserythroclados* is recorded in Argentina, Paraguay and southern Brazil.

### ﻿17. *Rubusfloribundus* Kunth, Nov. Gen. Sp. [H.B.K.] 6[Quarto]: 219. 1823.

≡ Rubusjamaicencisvar.floribundus (Kunth) Kuntze, Revis. Gen. Pl. 1: 221. 1891. ≡ *Rubusabundus* Rydb., N. Amer. Fl. 22(5): 454. 1913, nom. illeg. superfl.

= *Rubusrobustus* C.Presl, Epimel. Bot.: 196. 1851. • Type. Peru. “In vallibus cordillerum Peruviae” [in the valleys of the Peruvian cordillera], *T. Haenke s.n.* (lectotype, designated by Romoleroux 1996, pg. 45: PRC-450067 [image!]).

= Rubusfloribundusvar.nimbatus J.F.Macbr., Publ. Field Mus. Nat. Hist., Bot. Ser. 8: 118. 1930, syn. nov. ≡ Rubusrobustusvar.nimbatus (J.F.Macbr.) J.F.Macbr., Publ. Field Mus. Nat. Hist., Bot. Ser. 13, pt. 2: 1101. 1938. • Type. Peru. Huánaco: Huacachi, near Muña, ca 6500 ft, 20 May–01 Jun 1923 (fl, fr), *J.F. Macbride 3894* (holotype: F-V0041909F [image!]).

**Type. Ecuador. [Loja**]: “Crescit in andibus loxensium” [grows on Loja´s Andes], “800–1800 hex”, Aug (fl), *A. von Humboldt & M.A. Bonpland 3396* (lectotype, designated by Romoleroux 1996, pg. 42: P-00679379 [image!]; isolectotype: P-00162116 [image!]).

**Nomenclature notes.**Romoleroux (1996) cited the lectotype of *R.floribundus* Kunth at P, but we located several additional sheets at P that agreed with the information provided in the protologue. The situation is similar to that of *R.bogotensis*, where the lectotype has a label from the “Herbier Humboldt & Bonpland”. We cite the specimen P-00162116 as an isolectotype and specimen P-00162114 as possible original material because it lacks the collection number. The name *Rubusfloribundus* Weihe has been incorrectly used in Colombia, Peru and Bolivia for several years. The basis of the problem began with Rydberg, who cited the publication of *R.floribundus* Weihe & Nees as dated by 1821 and treated this species name on the basis of erroneously presumed priority. He then used the name *R.abundus* to replace *R.floribundus* Kunth (Rydberg 1913). However, the correct publication date of *R.floribundus* Weihe is 1825, and on the basis of priority the legitimate name is *R.floribundus* Kunth, as cited by Romoleroux (1996).

**Taxonomic notes.** The synonymy follows the revision of Romoleroux (1996), but we also recognize R.floribundusvar.nimbatus as a synonym of *R.floribundus* due to the lack of clear morphological characters to distinguish it from the type of *R.floribundus*.

**Possible original material of *Rubusfloribundus* Kunth: Ecuador. Loja**: “Andes” *A. von Humboldt & M.A. Bonpland s.n.* (P-00162114 [image!]).

**Specimens examined. Ecuador. • Loja**: Carretera Yangana-Cerro Toledo, 04°20.000'S, 79°08.000'W, 1970–2450 m, 14 Nov 1990 (fl), *K. Romoleroux 1160B* (QCA-137902). **Peru. • Cajamarca**: Agua Tapada, 07°10.142'S, 78°31.768'W, 3072 m, 21 May 2013 (fr), *I. Sánchez, E. Lineares & A. Galán 3205* (CPUN-23561, USM-289503). **Bolivia. • Santa Cruz**: Caballero, 15–25 km al N de San Juan de Potrero, hacia Cerro Bravo, 17°48.000'S, 64°15.000'W, 2000–2500 m, 6 Jun 1992 (fl), *T. Killeen & I. Vargas 4067* (MO-1604562).

**Distribution.***Rubusfloribundus* is recorded along the Andes of southern Ecuador, Peru and Bolivia.

### ﻿18. *Rubusgeoides* Sm., Pl. Ic. Ined.: t. 19 (as “XIX”). 1789.

≡ *Dalibardageoides* (Sm.) Pers., Syn. Pl. [Persoon] 2(1): 53. 1807. ≡ *Rubusantarcticus* Kuntze, Meth. Sp.-Beschr. Rubus 115. 1879, nom. illeg. superfl.

**Type. Chile.** Magellan, *P. Commerson s.n*. (holotype: LINN-HS-902.95 [image!]).

**Nomenclature notes.** We traced back the collection LINN-HS-902.95, which corresponds to the drawing of *R.geoides* cited in the protologue. According to Art. 9.1 of the ICN, we recognize this specimen as the holotype of *R.geoides*. Kuntze (1879) proposed *R.antarcticus* to unite *R.geoides*, *R.radicans* and *R.gunnianus* in one species. Therefore, in the diagnosis and description of *R.antarcticus*, characters of these three taxa are mixed. However, *R.geoides*, *R.radicans* and *R.gunnianus* are separate, distinct species. *Rubusantarcticus* is therefore an illegitimate, superfluous name (ICN 52.1), since the oldest, legitimate name (*R.geoides*) should have been accepted in its place.

**Taxonomic notes.** We recognize *R.geoides* and *R.radicans* as separate species on the basis of the short pedicels (> 1.5 cm) and glabrous leaves of *R.geoides* compared to the long pedicels (> 2 cm) and pubescent leaves of *R.radicans*.

**Specimens examined. Argentina. • Chubut**: Lago Futalaufquen, 27 Mar 1949 (fr), *T.M. Pedersen 313* (US-03733386). • **Tierra del Fuego**: Jan 1769 (fl), *J. Banks & D. Solander s.n.* (US-01299814); Estancia Harberton, 26 Nov 1967 (fr), *N. Goodall 1027* (US-03733384). **Chile. • [Valparaíso**]: Archipiélago Juan Fernández, “Masafuera” [Isla Alejandro Selkirk], Las Torres, 1100 m, 28 Jan 1955 (fl) *I. Skottsberg & C. Skottsberg 158* (US-03733381); same locality as for preceding, 1150–1300 m, 14–25 Feb 1917 (fr), *I. Skottsberg & C. Skottsberg 404* (US-03733382); Archipiélago Juan Fernández, “Masafuera” [Isla Alejandro Selkirk], Los Inocentes, 1000–1200 m, 02 Dec 1965 (fl, fr), *O.T. Solbirg, H.E. Moore & J. Walker 3740* (US-03733380).

**Distribution.***Rubusgeoides* is recorded in southern Argentina and the Tierra del Fuego Archipelago in Argentina and Chile.

### ﻿19. *Rubusglabratus* Kunth, Nov. Gen. Sp. [H.B.K.] 6[Quarto]: 221. 1823.

= *Rubusostrinus* Focke, Biblioth. Bot. 18, Heft 83: 21. 1914, syn. nov. • Type. Bolivia. [La Paz]: Nord-Yungas, 4000 m, *O. Buchtien 2857* (lectotype, designated here: US-00641902 [image!]).

**Type. Colombia.** “Pasto, prope Guachucal” [near Guachucal], “1620 hex”, Dec (fl), *A. von Humboldt & M.A. Bonpland 2187* (lectotype, designated by Romoleroux 1996, pg. 17: P-00679382 [image!]; isolectotypes: B-10-0248188 [image!], P-00162117 [image!], P-00162119 [image!]).

**Nomenclature notes.** The situation of the typification of *R.glabratus* is similar to that of *R.bogotensis* and *R.floribundus*. Romoleroux (1996) cited the lectotype at P, but we found several sheets corresponding to the information provided in the protologue. The lectotype of *R.glabratus* has a label from the “Herbier Humboldt & Bonpland”. Focke (1914) cited the collection *Buchtien 2857* in the protologue of *R.ostrinus* and we designate the specimen at US as the lectotype of this name (Art. 9.3, 9.11 and 9.12 of the ICN).

**Taxonomic notes.** We recognize *R.ostrinus* as a new synonym of *R.glabratus* due to the absence of trichomes in branches and leaves, presence of small, trifoliolate leaves, asymmetrically ovate stipules, solitary flowers or small inflorescences with up to 5 flowers, and ovate sepals, with bi- or trifurcations towards the apex when mature present in both type collections.

**Specimens examined. Venezuela. • Mérida**: Sierra Nevada, Sep 1846 (fl), *N. Funck & L.J. Schlim 1142* (P-03340873) • **Trujillo**: Boconó, Parque Nacional Guaramacal, 2200–2900 m, 15 Jun 2001 (fl), *L.H. Dorr, S.M. Niño & R. Caracas 9024* (US-00662899). **Colombia. • Boyacá**: Páramo de la Rusia, NW de Duitama, 3755 m, 12 Dec 1972 (fl), *A.M. Cleef 7078* (U-1557324). • **Cauca**: Puracé, 3300 m, 19 Sep 2003 (fr), *J.P.M. Martínez 141* (COL-000049524). **Ecuador. • Carchi**: Tulcán-El Ángel road, ca 9 km SW of Pan American Highway, 00°48.000'N, 77°48.000'W, 3385 m, 04 Nov 1990 (fl), *J.L. Luteyn, J. Ballington, M. Thompson, K. Romoleroux & R. Castillo 14030* (QCA-91984). • [**Loja**]: “Loxa”, *A. von Humboldt & M.A. Bonpland 2187* (P-000162118). **Peru. • Cusco**: Paucartambo, Acnajaco, subida a Quellhua Ccocha, Parque Nacional Manu, 3404 m, 01 Jul 1991 (fl, fr), *A. Cano & S. Baldeón 4912* (USM-108815). **Bolivia. • La Paz**: Franz Tamayo, Parque Nacional Madidi, Munagamachay, entre Keara y Mojos, 14°41.567'S, 69°00.467'W, 3404 m, 24 Jun 2005, *A. Fuentes, T. Miranda, J. Colque, R. Hurtado, I. Jímenez, E. Cuevas & R. Cuevas 8652A* (QCA-91958). • **Cochabamba**: Chaparé, km 104 on raod from Cochabamba to Chaparé and Villa Tunaré, 3025 m, 17 Feb 1971 (fl, fr), *J.G. Hawkes, J.P. Hjerting, P.J. Cribb & Z. Huamán 4441* (MO-1604544).

**Distribution.***Rubusglabratus* is recorded along the Andes of Venezuela, Colombia, Ecuador, Peru and Bolivia.

### ﻿20. *Rubusglaucophyllus* Vargas, Revista Univ. (Cuzco) 32(84): 263. 1943.

**Type. Peru. Cusco**: La Convención, alturas de Quillabamba, “El Dorado”, 2900 m, 03 Aug 1943 (fl, fr), *C. Vargas 3502* (holotype: CUZ-4002!; isotype: CUZ-4003!).

**Additional specimens examined. Peru. • Cusco**: Paucartambo, Jamamayo, 1800 m, 04 May 1947 (fl), *C. Vargas 6488* (CUZ-5607!).

**Distribution.***Rubusglaucophyllus* is known only from the type collection.

### ﻿21. *Rubusglaucus* Benth., Pl. Hartw. [Bentham]: 173. 1845.

**Type. Ecuador. [Pichincha**]: “In declivitate montis Pichincha, et frequenter cultus sub nomine Mora de Castillo” [in slopes of Pichincha mountain, and frequently cultivated under the name “Mora de Castilla”], *T. Hartweg 973* (lectotype, designated by Romoleroux 1996, pg. 29: K-000424919 [image!]; isolectotype: LD-1036950 [image!]).

**Nomenclature notes.** The situation of the typification of *R.glaucus* is similar to that of *R.compactus*, where the original material was available at two herbaria. We located specimens from *Hartweg 973* at K and LD, both of which correspond to the protologue information. Considering that Romoleroux (1996) cited the holotype in K, according to Art. 7.11 of the ICN, this was an effective typification at that time. Therefore, we here correct the designation to lectotype.

**Specimens examined. Colombia. • Boyacá**: Paipa, Vereda La Pradera, 2650 m, 02 Dec 1978 (fl, fr), *S. Díaz 1445* (COL-000197464). **Ecuador. • Carchi**: Tulcán-Maldonado road, 3–10 km of Tulcán, 00°50.000'N, 77°50.000'W, 2954–3077 m, 02 Nov 1990 (fl, fr), *J.L. Luteyn, J. Ballington, M. Thompson, K. Romoleroux & R. Castillo 104018* (QCA-92020, QCA-7000025).

**Distribution.**Romoleroux et al. (2014) cited *Rusby 471* (Bolivia) as *R.glaucus*, but we re-identified it as *R.ruizii*. No other collections of *R.glaucus* from Bolivia are known. The “Mora de Castilla”, *R.glaucus*, is distributed from Mexico through Central America, finding its southern range limit in the Andes of Ecuador.

### ﻿22. *Rubusguyanensis* Focke, Abh. Naturwiss. Vereins Bremen 4: 160. 1874.

≡ “*Rubusschomburgkii* Klotzsch”, Reis. Br.-Guiana [Ri. Schomburgk] 3: 1102. 1848, nom. nud.

**Type. Guyana.** Roraima, Nov 1842 (fl), *R. Schomburgk 1038* (lectotype, designated here: US-00468755 [image!]; isolectotype: NY-64737 [image!]).

**Nomenclature notes.**Focke (1874) published *R.guyanensis*, presumably based on the same specimen cited by Klotzch (Schomburgk et al. 1848) as “*R.schomburgkii*”. Klotzch’s name, despite being older, is invalid because it lacks a diagnosis or description (Art. 38.1 of the ICN) and does not refer to a description in an earlier publication (Art. 38.13). Furthermore, in the protologue, Focke (1874) cited the specimen from *R. Schomburgk* without indicating a specific herbarium. However, we found five specimens with the correct locality and collector, but with different collection numbers, that can be divided into three groups:

1. *R. Schomburgk 1038* at US and NY;
2. *R. Schomburgk 688* in P (two collections);
3. *R. Schomburgk 688(1038)* in K.

The collections in group 1 had a label from herbarium B (Ex Museo botanico Berolinense), the same collection year and locality as in the protologue, while those in groups 2 and 3 had only the same locality. Furthermore, the specimen in group 3 had two collection numbers. Considering that Focke was working at B at the time of the description, the original material must have been housed at B at some stage. We therefore designate the specimen at US as the lectotype, and the leaflet, flower fragment and photograph at NY is an isolectotype. The specimens in groups 2 and 3 are treated here as possible original material.

**Possible original material of *Rubusguyanensis* Focke: Guyana.** Roraima, *R. Schomburgk 688* (P-03372098 [image!], P-04114169 [image!]); same locality as for preceding, *R. Schomburgk 688(1038)* (K-000424881 [image!]).

**Specimens examined. Venezuela. • Trujillo**: Boconó, Parque Nacional Guaramacal, 1800–1900 m, Aug 2000 (fl), *B. Stergios & R. Caracas 18745* (US-00628291); same locality as for preceding, 09°15.233'N, 70°11.368'W, 2677–3100 m, 14 Jun 2001 (fr), *L.J. Dorr, S.M. Miño & R. Caracas 8974* (US-00662896). **Colombia. • Huila**: Balsilla, on Río Balsillas, 2000–2300 m, 03–06 Aug 1917 (fl), *H.H. Rusby & F.W. Pennell 912* (US-03733450). **Brazil. • Roraima**: “am Abhang der Felsen” [on the rock precipice], 2200 m, Dec 1909 (fl), *E. Ule 8617* (L-1907464).

**Distribution.***Rubusguyanensis* is recorded in Colombia, Venezuela, Guyana and northern Brazil.

### ﻿23. *Rubusimperialis* Cham. & Schltdl., Linnaea 2(1): 13. 1827.

**Type. Brazil. Santa Catarina**: Apúna, Faxinalzinho, 27°10.817'S, 49°23.617'W, 793 m, 27 Sep 2018 (fl), *A. Kassner-Filho, D. Santos, G. Bollmann & L.F. Althoff 3519* (neotype, designated here: FURB-60544 [image!]).

**Nomenclature notes.**Schlechtendal and Chamisso (1827) cited five specimens in the protologue: “St. Catharina” and “Rio de Janeiro” collected by the authors, “prope Clemente ad Rio Paquaquer” collected by Beyrich, and “in provinciis Rio grande do Sul” and “Montevideo” collected by Sellow. However, we were unable to locate any of the syntypes. *Rubusimperialis* is a well-documented species from Brazil, thus we studied the material available and compared it with the description. We provisionally accept this species. As there is no extant original material, according to Art. 9.8 of the ICN, we designate *A. Kassner-Filho 3519* as the neotype of *R.imperialis*.

**Specimens examined. Brazil. • Paraná**: Barras, 04 Jan 1974 (fr), *G. Hatschbach 33628* (MBM-034288). **Argentina. • Tucumán**: En los matorrales de Tucumán, 8 Oct 1887 (fl), *L. 122* (P-03373467).

**Distribution.**Romoleroux et al. (2014) cited *Solomon 18420* (Bolivia) as *Rubusimperialis*, but this specimen was re-evaluated here as *R.boliviensis*. Therefore, *R.imperialis* is found only in Argentina and Brazil.

### ﻿24. *Rubusjamaicensis* L., Syst. Nat., ed. 12. 2: 349. 1767.

≡ “Rubusjamaicensisvar.normalis Kuntze”, Revis. Gen. Pl. 1: 221. 1891, nom. inval.

= Rubusjamaicensisvar.nudicaulis Kuntze, Revis. Gen. Pl. 1: 221. 1891, syn. nov. • Type. Venezuela. May 1874, *O. Kuntze s.n.* (lectotype, designated here: NY-429644 [image!]).

**Type.** (lectotype, designated by Adams in Cafferty and Jarvis 2002, pg. 543): [icon] “*Rubus foliis longioribus subtus molli lanugine obductis & incanis, flore & fructu minoribus*” in Sloane, Voy. Jamaica 2: t. 213, f. 1. 1725. • **Epitype. Jamaica.***H. Sloane s.n.* (epitype, designated here: BM-000594064 [image!]).

**Nomenclature notes.** Adams (Cafferty and Jarvis 2002) cited an illustration as the lectotype of *R.jamaicensis*. At the same time, he cited the specimen BM-000594064 as the “topotype”. This collection was used for the illustration, so we select it as the epitype of this name. The original illustration is available at BM (BM-000594063).

Kuntze (1891) used “R.jamaicensisvar.normalis” to distinguish the typical form of *R.jamaicensis* from three other varieties, thus including its type. According to Art. 24.3 of the ICN, “R.jamaicensisvar.normalis” is an invalid name because its final epithet differs from that of the corresponding higher taxon. On the other hand, the other name R.jamaicensisvar.nudicaulis is legitimate. We located three specimens at NY with handwritten annotation by Kuntze and designate specimen NY-429644 as the lectotype of this name. The other two specimens are treated here as original material.

**Taxonomic notes.** We consider R.jamaicensisvar.nudicaulis to be a synonym of *R.jamaicensis*. It has the same curved, abundant prickles on the petioles, the same indument of petioles and leaves, especially the pannose abaxial surface of the leaf, and the same absence of or very few trichomes on the branches as in the epitype of *R.jamaicensis*.

**Original material of Rubusjamaicensisvar.nudicaulis Kuntze: Venezuela.** May 1874, *O. Kuntze s.n.* (NY-990592 [image!], NY-990593 [image!]).

**Specimens examined. Guyana. • Cuyuni-Mazaruni**: Mount Maingma, southern slopes of summit escarpment, Arabaru River, 05°12.277'N, 60°34.598'W, 1360 m, 11 Jun 2014 (fr), *H.D. Clarke, C. Perry, E. Tripp, S. Stern & D. Gittens 11572* (US-00889792). **Venezuela. • Miranda**: Oripoto, 1400 m, 16 Jan 1954, *Bro. Gines 4564* (US-03733828). **Colombia. • Norte de Santander**: Northern slope of Mesa de los Santos, 1000–1500 m, 11–15 Dec 1926 (fr), *E.P. Killip & A.C. Smith 15021* (US-03733613).

**Distribution.***Rubusjamaicensis* is reported from Jamaica, northern Guyana, northern Venezuela and northern Colombia.

### ﻿25. *Rubuskillipii* A.Berger, J. Wash. Acad. Sci. 16(6): 161. 1926.

= *Rubuspastasanus* Diels, Notizbl. Bot. Gart. Berlin-Dahlem 15: 370. 1941. • Type. Ecuador. Pastaza: Río Pambay, a lado del paso lateral de Puyo, 01°28.089'S, 78°00.542'W, 955 m, 12 Aug 2023, *D. Espinel-Ortiz & C. Restrepo 391* (neotype, designated here: QCA!; isoneotype: BONN! four sheets).

**Type. Colombia. Cauca**: “La Gallera”, Micay Valley, Cordillera Occidental, clearing near Río San Joaquin, 1100–1300 m, 29–30 Jun 1922, *E.P. Killip 7835* (holotype: US-1142423 [image!]; isotypes: GH-40520 [image!], NY-429646 [image!]).

**Notes.**Diels (1941) cited *H. Schultze-Rhonhof 2969* as the holotype of *Rubuspastasanus*, which was in B (Renner 1993). It seems to have been destroyed during WWII, and no duplicates have been located (Romoleroux 1996). As no original material is extant, based on Art. 9.16 of the ICN, we designate collection *D. Espinel-Ortiz & C. Restrepo 391* as the neotype of *R.pastasanus*. It was collected in Puyo (near Mera) at the same altitude and matches the original description.

**Specimens examined. Ecuador. • Tungurahua**: Vía a Baños entre Río Negro y Mera, a 2 km de Río Negro en carretera E30, 01°25.194'S, 78°10.536'W, 1333 m, 12 Aug 2023, *D. Espinel-Ortiz y C. Restrepo 390* (QCA).

**Distribution.***Rubuskillipii* is found in southern Colombia and Ecuador.

### ﻿26. *Rubuslaegaardii* Romol., Fl. Ecuador 56: 15. 1996.

**Type. Ecuador. Morona Santiago**: Near the pass on the road Sigsig-Gualaquiza, 3°09.000'S, 78°43.000'W, 3300 m, 29 May 2019, *S. Lægaard, L.G. Clark & P. Stern 103042* (holotype: AAU [image!] two sheets; isotype: QCA-92046!).

**Specimens examined. Ecuador. • Loja**: Carretera Yangana-Zumba, desvío Cerro Toledo, 04°22.485'S, 79°06.677'W, 3080 m, 09 Feb 2002 (fl, fr), *K. Romoleroux, S. León-Yánez & V. Sandoya 4075* (QCA-92062, QCA-7000123). **Peru. • Cajamarca**: San Ignacio, abaconas, Santuario Nac, Tabaconas-Namballe, alrededores de las lagunas Coyona (Arrebiatadas), 05°13.525'S, 79°16.480'W, 3140–3180 m, 08 Apr 2003 (fl), *S.M. Baldeón-Malpartida & L.A. Ocupa 5126* (USM-266692).

**Distribution.***Rubuslaegaardii* is known from the Andes of southern Ecuador and northern Peru. We report here for the first time the presence of *R.laegaardii* in Peru.

### ﻿27. *Rubuslongistipularis* Espinel-Ortiz & Romol., PhytoKeys 187: 143. 2021.

**Type. Ecuador. Pichincha**: Nono-Tandayapa road, between km 116–117, 00°01.787'S, 78°38.567'W, 1950 m, 26 Jul 2021 (fl, fr), *D. Espinel-Ortiz & H.G. Abad 281* (holotype: QCA-243418!, QCA-7010714! to QCA-7010723!); isotypes: BONN! four sheets, HA-13781!, HUTI!, QAP!).

**Specimens examined. Ecuador. • Pichincha**: Quito, Nanegalito, vía a San Tadeo, Área Protegida Privada Bellavista, 00°01.011'S, 78°40.867'W, 2255 m, 03 Dec 2021 (fr), *D. Espinel-Ortiz y H.G. Abad 297* (QCA-244016) • same locality as for preceding, 00°01.938'S, 78°41.756'W, 2303 m, 03 Dec 2021 (fr), *D. Espinel-Ortiz y H.G. Abad 299* (QCA-244018, QCA-7010803, QCA-7010804).

**Distribution.***Rubuslongistipularis* is reported from the Andes of northern and central Ecuador.

### ﻿28. *Rubusloxensis* Benth., Pl. Hartw. [Bentham]: 128. 1843.

= *Rubusextensus* Fritsch in Szyszyl., Diss. Cl. Math.-Phys. Acad. Litt. Cracov. 29: 221. 1894. • Type. Peru. Chonta Cruz, Tambillo, *C. von Jelski 3* (lectotype, designated by Romoleroux 1996, pg. 7: B-10-0248186 [image!]).

= Rubusextensusf.major Fritsch in Szyszyl., Diss. Cl. Math.-Phys. Acad. Litt. Cracov. 29: 221. 1894. • Type. Peru. Cutervo, *C. von Jelski 4* (lectotype, designated by Romoleroux 1996, pg. 7: KRA (n.v.)).

**Type. Ecuador. [Loja**]: “In montibus prope Loxa” [In mountain near Loja], *T. Hartweg 731* (lectotype, designated by Romoleroux 1996, pg. 7: K-000424878 [image!]; isolectotype: LD-1211845 [image!]).

**Nomenclature notes.** The situation of the typification of *R.loxensis* is similar to that of *R.compactus* and *R.glaucus*. Specimens from *Hartweg 731* are available at K and LD, and a lectotype is required (Art. 9.3 of the ICN). Romoleroux (1996) effectively typified the species name at that time (Art. 7.11) when she cited the collection in K as the holotype. We correct the type status to lectotype.

**Taxonomic notes.** The synonymy follows the revision of Romoleroux (1996).

**Specimens examined. Ecuador. • Loja**: Yangana-Cerro Toledo, 04°23.000'S, 79°07.000'W, 2900 m, 30 Jan 1999 (fl, fr), *S. Lægaard 19518* (QCA-92058). **Peru. • Cajamarca**: Jaén, Sallique, La Cocha, 05°40.967'S, 79°14.883'W, 2960 m, 21 Jun 1998 (fr), *J. Campos, C. Díaz, H. Tineo & P. Julca 5054* (USM-163652).

**Distribution.***Rubusloxensis* is recorded in the Andes of southern Ecuador, Peru and Bolivia (Romoleroux et al. 2014).

### ﻿29. *Rubusmandonii* Focke, Abh. Naturwiss. Vereins Bremen 4: 162. 1874.

**Type. Bolivia. La Paz**: Larecaja province, 2750–3200 m, Oct 1858–Apr 1859 (fl, fr), *G. Mandon 659* (lectotype, designated here: S-08-15139 [image!]; isolectotypes: BR-33828558 [image!], K-000424920 p.p. [image!], NY-435731 [image!], P-03372114 p.p. [image!], P-00682384 [image!]).

**Nomenclature notes.**Focke (1874) cited *Mandon 659* at LUB in the protologue of *R.mandonii*. However, we could not locate it, but found duplicates at BR, K, NY, P and S. According to Art. 9.3, 9.11, and 9.12 of the ICN, we designate the specimen from S as the lectotype, because it is the only one with 5-foliolate leaves corresponding to the original description. All the other specimens are treated as isolectotypes, except for specimen BR-33828558, which has a different locality and is treated here as possible original material.

**Taxonomic notes.***Rubusmandonii* is similar to *R.nubigenus*, but differs by the glabrous or slightly pubescent branches, narrowly elliptic stipules, and 5-foliolate leaves of *R.mandonii* vs. the tomentose branches, broadly ovate to auriculate stipules and trifoliate leaves of *R.nubigenus*.

**Specimens examined. Bolivia. • Cochabamba**: Siberia, 3300 m, Jul 1955 (fl), *M. Cárdenas 5215* (L-1921409).

**Distribution.***Rubusmandonii* is recorded in northern and central Bolivia.

### ﻿30. *Rubusmaquipucunensis* Espinel-Ortiz & Romol., PhytoKeys 187: 149. 2021.

**Type. Ecuador. Pichincha**: cantón Quito, parroquia Nanegal, in front of the Ecological Reserve Maquipucuna entrance, 00°07.457'S, 78°37.744'W, 1278 m, 11 Feb 2021 (fl, fr), *D. Espinel-Ortiz, C. Restrepo & A. Sanguano 269* (holotype: QCA-243282!, QCA-7010670! to QCA-7010679!; isotypes: HA-13781!, HUTI!, LOJA!, Q!, QCNE!).

**Specimens examined. Ecuador. • Santo Domingo de los Tsáchilas**: old road San Juan-Chiriboga, km 60–70, 00°17.000'S, 78°50.000'W, 1000–1500 m, 09 Jan 1993 (fl), *K. Romoleroux & A. Freire 1514* (QCA-92036, QCNE-77110).

**Distribution.***Rubusmaquipucunensis* is recorded in the western flank of the Andes of northern Ecuador.

### ﻿31. *Rubusmegalococcus* Focke, Abh. Naturwiss. Vereins Bremen 4: 157. 1874.

= *Rubusbuchtienii* Focke, Repert. Spec. Nov. Regni Veg. 9: 237. 1911, syn. nov. • Type. Bolivia. La Paz: Nor Yungas, Unduavi, 3300 m, Nov 1910 (fl), *O. Buchtien 641* (lectotype, designated here: NY-429634 [image!]).

**Type. Bolivia. La Paz**: Larecaja, “viciniis Sorata” [near Sorata], 3000–3200 m, 1859 (fl), *G. Mandon 662* (lectotype, designated by Romoleroux 1996, pg. 31: W (n.v.); isolectotypes: K-000424917 [image!], P-00682383 [image!], P-00682386 [image!]).

**Nomenclature notes.**Focke (1911a) cited *Buchtien 641* in the protologue of *R.buchtienii*, but did not specify a herbarium. We located a single specimen at NY and, according to Art. 9.3, 9.11, and 9.12 of the ICN, we designate it as the lectotype.

**Taxonomic notes.** We consider *R.buchtienii* to be a synonym of *R.megalococcus* because it has the same glabrous or puberulent indument in almost the whole plant, glabrous adaxial surface of the leaf with some trichomes near the veins, axillary inflorescences with up to 25 flowers and ovate-lanceolate sepals as *R.megalococcus*.

**Specimens examined. Ecuador. • Loja**: Parque Nacional Yacuri, Jimbura, 04°42.950'S, 79°25.233'W, 3230–3450 m, 22 Apr 2015 (fr), *Á.J. Pérez, N. Zapata, W. Santillán & R. Jiménez 8681* (QCA-233883). **Peru. • Pasco**: Oxapampa, Huancabamba, zona de amortiguamiento del Parque Nacional Yanachaga Chemillén, 10°19.083'S, 75°36.467'W, 2567 m, 25 Jun 2008 (fr), *A. Monteagudo, A. Peña, J.L. Mateo & R. Rivera 16499* (USM-234511). **Bolivia. • La Paz**: Nor Yungas, Unduavi, 3300 m, Nov 1910 (fr), *O. Buchtien 173* (B-10-0278021, BR-13347901, E-00296709, GH-40519, K-000042918, M-0214192, NY-429633); Sud Yungas, 1.8 km W of Unduavi on road to La Paz, 16°18.000'S, 67°55.000'W, 3300 m, 21 Mar 1984 (fr), *J.C. Solomon, B. Stein & M. Uehling 11970* (US-3733542).

**Distribution.***Rubusmegalococcus* is recorded in the Andes of southern Ecuador, Peru and Bolivia.

### ﻿32. *Rubusmollifrons* Focke, Repert. Spec. Nov. Regni Veg. 9: 236. 1911.

**Type. Colombia. Magdalena**: Santa Marta, 1898–1901 (fl), *H.H. Smith 2512* (neotype, designated here: US-03733639 [image!]).

**Nomenclature notes.** The name *R.mollifrons* has not been used for several years, due to the absence of a type specimen. In the protologue, Focke (1911a) did not cite any collection, but mentioned that *R.mollifrons* resembles *R.floribundus* and is found in the Caribbean near South America, in Colombia and Venezuela. Fortunately, he published a photograph of the original material in his later monograph on the genus *Rubus* (Focke 1914), but we were unable to locate any extant material. We therefore used the photograph of *Karsten 21* to identify the species and select *Smith 5212* as the neotype of *R.mollifrons* (Art. 9.8 of the ICN).

**Photograph of original material of *Rubusmollifrons* Focke: Colombia.** 1846 (fl), *H. Karsten 21* (F [image!]).

**Specimens examined. Venezuela. • Distrito Capital**: Parque Nacional El Ávila, camino hacia “El Paraíso”, 1100–1500 m, 29 Jul 1970 (fl), *R. Labbiente 74* (US-03733604); Galipan, Nov 1924 (fl), *A. Allart 168* (US-03733603); Las Flores, Sierra de El Ávila, 1600 m, 15 Dec 1938 (fl), *A.H.G. Alston 5509* (US-03733598).

**Distribution.***Rubusmollifrons* is reported from northern Venezuela and northern Colombia near the Caribbean.

### ﻿33. *Rubusneobrasiliensis* Espinel-Ortiz, Böhnert, Romol. & Weigend, nom. nov.

≡ “*Rubusbrasiliensis* Mart.”, Cat. Hort. Monac.: 173. 129. 1829, nom. nud. ≡ *Rubusbrasiliensis* Mart. ex Hook.f., Fl. Bras. (Martius) 14(2): 62. 1867, nom. illeg. superfl.

**Type. Brazil. Rio de Janeiro**: *C.F.P. von Martius s.n.* (lectotype, designated by Fuks 1984, pg. 16: M-0214193 [image!]).

**Nomenclature notes.** There are some problems regarding the correct nomenclature of widely used *R.brasiliensis*. This name first appeared in the book “Hortus Regius Monacensis”, where Martius used “*R.brasiliensis*”, but omitted its respective diagnosis and description (Schrank and Martius 1829). According to the Art. 38.1 of the ICN, descriptive matter was necessary for the name to be validly published. We could not find any reference to an earlier description (Art. 38.13), and later publications written by or edited by Martius referred to page 173 of “Hortus Regius Monacensis” (Martius 1843; Hooker 1867). Therefore, according to Art. 38 of the ICN, *R.brasiliensis* was not validly published in 1829 and 1843.

The next time this name appeared in a revision, was by Hooker (1867) in Flora brasiliensis. Here, Hooker (1867) included a description of the species and cited two gatherings: *Martius s.n.* and *Pohl s.n*. According to Art. 38 of the ICN this was a valid publication of *R.brasiliensis*, but the species name was illegitimate. In the protologue, Hooker (1867) included *R.organensis* as a variety of *R.brasiliensis*. Therefore, the correct name of the species should have been *R.organensis*. However, we consider *R.organensis* and *R.brasiliensis* to be two different taxa, so a replacement name is needed for the latter. We decided on *R.neobrasiliensis* as a replacement name of *R.brasiliensis*. The corresponding lectotype was effectively designated by Fuks (1984), when she treated *Martius s.n.* as the holotype (Art. 7.11).

**Taxonomic notes.** We follow the revision of Fuks (1984), but recognize *R.neobrasiliensis* and *R.organensis* as two distinct taxa due to the following differences. *Rubusneobrasiliensis* is characterized by straight prickles covering the whole plant, absent or rarely present stipitate glands, broader stipules (6–10 × 1.5–1.8 mm) and densely villous-tomentose, thick leaves, especially on the abaxial surface near the veins compared to the curved prickles and stipitate glands covering the whole plant, narrower stipules (9–10.5 × 0.9–1.1 mm) and villous leaves, especially near the veins on the adaxial and abaxial surface of *R.organensis*.

**Specimens examined. Brazil. • Bahia**: Abaíra, distrito de Catolés, caminho para o Pico do Barbado, Mata da Furquilha, 14 Apr 1999 (fl, fr), *R.C. Forzza, A.M. Amorim, S.C., D.E. Sant’ana & C.B. Costa 1216* (NY-1874351). **Paraguay. • Unknown**: *E. Hassler 4689* (NY-656597).

**Distribution.***Rubusneobrasiliensis* is recorded in southern Brazil and Paraguay.

### ﻿34. *Rubusnovogranatensis* Aspl., Bot. Not. 1939: 799. 1939.

**Type. Colombia. Cauca**: Cordillera Central, Puracé, 3700 m, Feb 1938 (fl), *K. von Sneidern 1794* (holotype: S-R-8022 [image!]; isotypes: A-40521 [image!], B-10-0248184 [image!], NY-429647 [image!]).

**Nomenclature notes.**Asplund (1939) cited the types of *R.novogranatensis* and *R.sneidernii*, but did not specify a herbarium. In the introduction, however, he stated that Kjell von Sneidern’s collections were deposited in S, where we located specimens corresponding to the information from the protologue. According to Art. 9.1 of the ICN, the specimens at S correspond to the holotypes of *R.novogranatensis* and *R.sneidernii*.

**Specimens examined. Colombia. • Valle del Cauca**: Cordillera Central, Quebrada Las Vegas, 3400–3500 m, 23 Mar 1946 (fr), *J. Cuatrecasas 20353* (U-01557318, US-03733836).

**Distribution.***Rubusnovogranatensis* is reported from the Cordillera Central in Colombia.

### ﻿35. *Rubusnubigenus* Kunth, Nov. Gen. Sp. [H.B.K.] 6[Quarto]: 220. 1823.

= *Rubusmacrocarpus* Benth., Pl. Hartw. [Bentham]: 129. 1844. • Type. Ecuador. Loja: “In jugo montium prope Loxa” [mountains near Loja], T. Hartweg 731* (lectotype, designated by Romoleroux 1996, pg. 22: K-000424922 [image!]; isolectotype: LD-1246215 [image!]).

= *Rubusstipularis* Benth., Pl. Hartw. [Bentham]: 173. 1845. • Type. Ecuador. Pichincha: “In decivitate montis Pichincha” [at the foot of Pichincha mountain], 11500 ft, *T. Hartweg 971* (lectotype, designated by Romoleroux 1996, pg. 22: K-000424924 [image!]; isolectotype: LD-1060102 [image!]).

= *Rubuslechleri* Focke, Abh. Naturwiss. Vereins Bremen 4: 161. 1874, syn. nov. ≡ Rubusroseusvar.lechleri (Focke) J.F.Macbr., Publ. Field Mus. Nat. Hist., Bot. Ser. 8: 118. 1930. • Type. Peru. Cajamarca: “In virgultis prope Agapta” [in bushes near Agapta], Jun 1854, *W. Lechler 1997* (lectotype, designated here: K-000424925 [image!]; isolectotype: GOET-010123 [image!]).

= *Rubusholtenii* Kuntze, Revis. Gen. Pl. 3[3]: 78. 1898, syn. nov. • Type. Bolivia. 2500 m, 1–4 Apr 1892 (fr), *O. Kuntze s.n.* (lectotype, designated here: NY-429640 [image!]).

= *Rubusandicola* Focke, Biblioth. Bot. 72: 36. 1910, syn. nov. • Type. Peru. Junín: Tarma, Huacapistana, 2600–2700 m, 20 Jan 1903 (fl, fr), *A. Weberbauer* (lectotype, designated here: B-10-1172570 [image!]).

= *Rubussneidernii* Aspl., Bot. Not. 1939: 799. 1939, syn. nov. • Type. Colombia. Cauca: “ad pag. El Tambo Munchique” [near El Tambo in Munchique], 2500 m, 26 Apr 1936 (fl), *K. von Sneidern 648* (holotype: S-R-8024 [image!]; isotypes: B-10-0248183 [image!], NY-429656 [image!]).

**Type. Ecuador. Pichincha**: Páramo de puntas, “1700 hex”, Jan (fl, fr), *A. von Humboldt & M.A. Bonpland 3088* (lectotype, designated by Romoleroux 1996, pg. 21: P-00679381 [image!]; isolectotype: B-W-09899-010 [image!], P-00162120 [image!]).

**Nomenclature notes.** The situation of the typification of *R.nubigenus* is similar to that of *R.bogotensis*, *R.floribundus* and *R.glabratus*. We located three sheets at P that were treated as type material of *R.nubigenus*, but only two of them correspond to the typification of Romoleroux (1996) and the information on the protologue. We omit specimen P-00162121 from the type material, although it has the same collection number, because it was collected in Peru, while the type of *R.nubigenus* was collected in Ecuador. The lectotype of *R.nubigenus* has a label from the “Herbier Humboldt & Bonpland”.

The situation of the typification of *R.macrocarpus* and *R.stipularis* is similar to that of *R.compactus*, *R.glaucus* and *R.loxensis*. We located the type material of these names at K and LD, so a lectotype is required for all of them (Art. 9.3 of the ICN). Romoleroux (1996) cited the holotypes of *R.macrocarpus* and *R.stipularis* at K, thus effectively typifying the names at that time (Art. 7.11); these type designations are here corrected to lectotypes.

We typify the other names cited here as synonyms of *R.nubigenus*, with the exception of *R.sneidernii*, whose holotype is at S (similar case as in *R.novogranatensis*). We designate *Lechler 1997* at K as the lectotype of *R.lechlerii*, and *Weberbauer 2281* as the lectotype of *R.andicola*. We located duplicates of Kuntze’s collections with his annotations in NY and US, and select specimen NY-429640 as the lectotype of *R.holtenii*, while the rest are treated here as original material.

**Taxonomic notes.** In addition to the previous synonyms for this name (Romoleroux 1996), we also consider *R.andicola*, *R.holtenii*, *R.lechleri* and *R.sneidernii* as synonyms of *R.nubigenus* and provide information on the typification of each name. The types of all these species have the same characters as the type of *R.nubigenus*: tomentose branches, orbicular stipules, trifoliolate leaves, abaxial leaf surface tomentose to deeply tomentose, ovate sepals, acute or acuminate apex, mostly bi- or trifurcate, and fruits with numerous, small drupelets.

**Original material of *Rubusholtenii* Kuntze: Bolivia. • Unknown**: Santa Rosa, 2500 m, 1892, *O. Kuntze s.n.* (US-00097920 [image!]); 2500 m, 1–4 Apr 1892, *O. Kuntze s.n.* (NY-429639 [image!], US-00097919 [image!]).

**Specimens examined. Colombia. • Magdalena**: Sierra Nevada de Santa Marta, 10°55.000'N, 73°57.000'W, 2500–2650 m, 04 Aug 1972 (fl), *J.H. Kirkbride, J. Forero & E. Forero 1869* (US-03733525). **Ecuador. • Imbabura**: Otavalo, vía a las lagunas de Mojanda, 00°09.267'N, 78°16.558'W, 3659 m, 24 Aug 2016, *D. Espinel-Ortiz, E. Bastidas & K. Romoleroux 12* (QCA-243403). • **Chimborazo**: Penipe, entrada oeste, sendero al Volcán Altar, 01°37.852'S, 78°29.810'W, 3524 m, 27 Nov 2019 (fl), *D. Espinel-Ortiz & C. Restrepo 197* (QCA-246102). **Peru. • Cusco**: Urubamba, Ollantaytambo, Garrapata, Pajonal, 13°04.950'S, 72°16.950'W, 3382 m, 25 Feb 2006 (fl, fr), *L. Valenzuela, J. Farfán, E. Suclli, I. Huamantupa & R. Ayerbe 6339* (CUZ, USM-231079). • **Unknown**: *A. von Humboldt & M.A. Bonpland 3088* (P-00162121). **Bolivia. • Santa Cruz**: Caballero. Laguna Brava, Cerro ponguillo, 17°47.892'N, 64°36.995'W, 2950–3085 m, 16 Apr 2003, *I.G. Vargas 6893* (QCA- 92202).

**Distribution.***Rubusnubigenus* is reported from the Andes of Colombia, Ecuador, Peru and Bolivia.

### ﻿36. *Rubusorganensis* Gardn., London J. Bot. 2: 342. 1843.

≡ Rubusbrasiliensisvar.organensis (Gardn.) Hook f., Fl. Bras. (Martius) 14(2): 62. 1867.

**Type. Brazil.** Organ Mountains [Serra dos Órgãos], 1836 (fl, fr), *G. Gardner 372* (lectotype, designated here: K-000424883 [image!]).

**Nomenclature notes.** The name *R.organensis* has not been typified, probably because it was consistently treated as a synonym of *R.brasiliensis* [= *R.neobrasiliensis*] from the middle of the 19^th^ century until recently (see *R.neobrasiliensis* notes) (Hooker 1867; Fuks 1984). Gardner (1843) cited number 372 from his personal collection in the protologue, and we found two specimens at K with this number on the label. However, although they refer to the same species and have the same collection number, they are different gatherings, collected in different years. According to Art. 9.4 of the ICN, we recognize both collections as original material examined by Gardner, and according to Art. 9.3, 9.11 and 9.12, we designate specimen K-000424883 as the lectotype of *R.organensis*. The other collection is treated here as original material.

**Original material of *Rubusorganensis* Gard.: Brazil.** Organ Mountains, 1838 (fl), *G. Gardner 372* (K-000424882 [image!]).

**Specimens examined. Brazil. • Paraná**: Prudentopolis, Relogio, 14 Apr 1964 (fl), *G. Hatschbach 11167* (US-01351265). • **Santa Catarina**: Canoinhas, Ruderal, W of Canoinhas on the road to Porto União, 750 m, 17 Dec 1956 (fr), *L.B. Smith & P.R. Reitz 8595* (US-01351269); 12 km N of Abelardo Luz, 26°32.000'S, 52°20.000'W, 900–1000 m, 08 Dec 1964 (fr), *L.B. Smith & R.M. Klein 13862* (US-01351263); Caçador, Ruderal, W of Caçador on the road to Taquara Verde, 900–1000 m, 23 Dec 1956 (fr), *L.B. Smith & P.R. Reitz 9096* (US-01351268). • **Minas Gerais**: Chacha Valley road, ca 2 km from Agricultural gate, 675 m, 21 May 1930 (fr), *Y. Mexia 4730* (US-01351276).

**Distribution.***Rubusorganensis* is recorded in southern Brazil.

### ﻿37. *Rubusparaguariensis* (Chodat & Hassl.) Basualdo & Zardini, Candollea 47 (Heft 2): 255. 1992.

≡ Rubushasslerivar.paraguariensis Chodat & Hassl., Bull. Herb. Boissier, ser. 2, 3: 799. 1903.

**Type. Paraguay. San Pedro**: “Jejui Guazu”, Sep 1899, *E. Hassler 4618* (holotype: G-00640009 [image!]).

**Distribution.** The species is found in Paraguay.

### ﻿38. *Rubuspendulus* Rusby, Torreya 33 (2): 41. 1933.

**Type. Colombia. Huila**: Balsillas, at Balsillas river, 2000–2100 m, 03–05 Aug 1917, *H.H. Rusby & F.W. Pennell 719* (holotype: NY-429649 [image!]).

**Specimens examined. Venezuela. • Táchira**: Cabecera del Río Quinimarí, arriba de las Quebradas Las Copas, 2500–2630 m, 11 Jan 1968 (fl, fr), *J.A. Steyermark, G.C.K. Dunsterville & E. Dunsterville 100727* (US-03733826, US-03733827). • **Yaracuy**: Sierra de Aroa, 9 km W of San Felipe, 10°21.000'N, 68°49.000'W, 900–1500 m, 5 Apr 1980 (fl), *R. Liesner & A. González 10061* (MBM-91017). **Colombia. • Huila**: Neiva, Vereda La Plata, Finca La Colonia (Antigua Carolina), 2000 m, 31 Oct 1996 (fl), *F. Llanos & W.F. Gerardino 2797* (COL-000197900). **Ecuador. • Pichincha**: Quito, Nanegalito, vía a San Tadeo, Área Protegida Privada Bellavista, 00°02.170'S, 78°42.067'W, 2297 m, 03 Dec 2021, *D. Espinel-Ortiz & H.G. Abad 300* (QCA-244065, QCA-7010819 to QCA-7010822). **Peru. • Piura**: Huancabamba, Rosario Alto, Cerro Pan de Azúcar, 04°55.900'S, 79°18.700'W, 2250 m, 03 Aug 1988 (fl, fr), *C. Díaz & H. Osores 3842* (USM-126985).

**Distribution.***Rubuspendulus* is reported from the Andes of Venezuela, Colombia, Ecuador and Peru.

### ﻿39. *Rubusperuvianus* Fritsch in Szyszyl., Diss. Cl. Math.-Phys. Acad. Litt. Cracov. 29: 220. 1894.

= *Rubushelioscopus* Focke, Bot. Jahrb. Syst. 54(1, Beibl. 117): 41. 1916, syn. nov. • Type. Peru. Ayacucho: Prov. Huanta, “Weg von Tambo über Osno zum Flusse Apurimac” [road from Tambo, via Osno, to river Apurimac], 3100–3400 m, 31 May 1910 (fl), *A. Weberbauer 5580* (holotype: B-10-0248187 [image!]).

= *Rubussparsiflorus* Focke ex J.F.Macbr., Publ. Field Mus. Nat. Hist., Bot. Ser. 8: 117. 1930, syn. nov. • Type. Peru. Huánaco: 9000 ft, 08–22 Jul 1922 (fl, fr), *J.F. Macbride & W. Featherstone 1674* (holotype: F-V0041932F [image!] p.p.; isotype: B-10-0248176 [image!]).

= Rubusbogotensissubsp.eglandulosus Killip, J. Wash. Acad. Sci. 24(1): 47. 1934, syn. nov. • Type. Colombia. Santander: Eastern slope of Páramo de Santurbán, toward Mutiscua, 3600–3900 m, 20 Feb 1927 (fl), *E.P. Killip & A.C. Smith 19595* (holotype: US-00097864 [image!]; isotypes: A-40516 [image!], GH-40517 [image!]).

**Type. Peru.** Cutervo, May 1879, *C. von Jelski 7* (lectotype, designated by Romoleroux 1996, pg. 34: KRA (n.v.)).

**Nomenclature notes.** The holotype of *Rubussparsiflorus* is mounted together with a 5-foliolate leaf that belongs to a different species, which was not previously recognized and may have caused confusion with its identification.

**Taxonomic notes.** We recognize *R.helioscopus*, *R.sparsiflorus* and R.bogotensissubsp.eglandulosus as synonyms of *R.peruvianus*. The types of *R.helioscopus* and *R.sparsiflorus* have the same fruits with large and few drupelets as is typical of *R.peruvianus*. They also have the same villous indument without stipitate glands covering the whole plant. The type of R.bogotensissubsp.eglandulosus has no fruit, but the villous indument without stipitate glands, long and narrow stipule, trifoliate leaves, and ovate sepals are the same as in *R.peruvianus*.

**Specimens examined. Colombia. • Santander**: Cordillera Oriental, páramo del Almorzadero, Peralonso, 3200 m, 19 Jul 1940 (fl), *J. Cuatrecasas & H. García-Barriga 9926* (COL-000197246, US-03733276). **Ecuador. • Azuay**: Vía Cuenca-Loja, a aprox. 500 m del desvío en la carretera E35, 03°14.208'S, 79°03.501'W, 3288 m, 13 Nov 2019, *D. Espinel-Ortiz & E. Bastidas-León 181* (QCA-246055). **Peru. • Cajamarca**: Hualgayoc, entre Quishuarani y Lares, 06°45.680'S, 78°36.018'W, 3523 m, 27 May 2014 (fl), *J. Montoya, E. Linares & A. Galán 3756* (USM-298276). **Bolivia. • La Paz**: Madidi, Apolobamba, Pelechuco-Río abajo, Santa Ana Ladera, 17°49.250'S, 69°04.017'W, 3547 m, 25 May 2009 (fl), *V. Torrez 572* (QCA-209074).

**Distribution.***Rubusperuvianus* is reported from the Andes of Colombia, southern Ecuador, Peru and Bolivia.

### ﻿40. *Rubusradicans* Cav., Icon. 5: 7. 1799.

≡ *Comaropsisradicans* (Cav.) Ser., Prodr. [A. P. de Candolle] 2: 555. 1825.

**Type. Chile.** “Ex San Carlos de Chiloe” [from San Carlos de Chiloé, now Ancud city], Feb (fl); *L. Née 931* (lectotype, designated here: MA-01-00476192!).

**Nomenclature notes.** We recognize *R.radicans* as a distinct species (see *R.geoides* notes) and provide information on its typification. Cavanilles (1799) described *R.radicans* with collections from *L. Née*, before they were deposited at MA. Based on the common name “*Rubusfrutilla*” mentioned in the protologue, we traced two specimens with different collection numbers: *Née 768* and *Née 931*, which have a handwritten annotation of “Rubus” and “frutilla”, respectively. The lectotype has flowers and fruits, and a label with full details from the original publication. The other collection is treated as original material of the name.

**Original material of *Rubusradicans* Cav.: Type. Chile.** “Ex San Carlos de Chiloe” [from San Carlos de Chiloé, now Ancud city], Feb (fl), *L. Née 768* (MA-01-00476191!).

**Specimens examined. Bolivia. • Cochabamba**: Chapare, Abro de Colomi, 13500 ft, 16 Mar 1939 (fl, fr), *E.K. Balls 6285* (US-00641905). **Argentina. • Río Negro**: Puerto Blest, Nov 1926 (fl), *R.C. Shannon & E.S. Shannon 20* (US-03733386). **Chile. • Los Lagos**: Puerto Montt, Nov 1925 (fl, fr), *F. Claude-Joseph 3294* (US-03733392). • **Los Ríos**: Valdivia, Panguipulli, 200 m, Oct 1926 (fl), *P.A. Hollermayer 1393* (US-03733566, US-03733379, Z-282091). • **Unknown**: Quitratúe, *F. Claude-Joseph 5870* (US-03733390); *R.A. Phillippi 799* (US-03733388).

**Distribution.***Rubusradicans* is reported from central Bolivia, southern Argentina and southern Chile.

### ﻿41. *Rubusroseus* Poir., Encycl. [J. Lamarck & al.] 6(1): 245. 1804.

= *Rubusrosiflorus* Hook., Icon. Pl. 1: t. 46 (XLVI). 1837 (as “*rosaeflorus*”), syn. nov. ≡ Rubusroseusvar.rosiflorus (Hook.) Focke, Biblioth. Bot. 72: 36. 1910 (as “*rosaeflorus*”). • Type. Ecuador. Pichincha: Woods on the western declivity of Pichincha, 9000 ft, *W. Jameson 101* (holotype: K-000424926 [image!]).

= *Rubussantarosensis* Kuntze, Revis. Gen. Pl. 3[3]: 80. 1898, syn. nov. ≡ Rubusroseusvar.santarosensis (Ktze.) J.F.Macbr., Publ. Field Mus. Nat. Hist., Bot. Ser. 8: 118. 1930. • Type. Bolivia. Santa Rosa, 3000 m, 01–04 Apr 1892 (fr), *O. Kuntze s.n.* (lectotype, designated here: NY-429655 [image!]).

= *Rubuslloensis* Benoist, Bull. Soc. Bot. France 81(2): 325. 1934. • Type. Ecuador. Pichincha: Palmira, 12 Feb 1931 (fl), *R. Benoist 3842* (lectotype, designated by Romoleroux 1996, pg. 19: P-00162127 [image!], isolectotype: P-00162128 [image!]).

= Rubusnubigenusvar.subinermis Benoist, Bull. Soc. Bot. France 90: 15. 1943, syn. nov. • Type. Ecuador. [Pichincha]: “Pentes orientales du Mojanda” [in western slopes of Mojanda], 03 Mar 1931 (fl), *R. Benoist 4000* (holotype: P-03145738 [image!]).

**Type. Peru.***J. Dombey s.n.* (holotype: P-00678395 [image!]).

**Nomenclature notes.** The situation of the typification of *R.roseus* is similar to that of *R.coriaceus*, where Poiret (1804) cited a collection from the Jussieu Herbarium. We found one specimen with a label from the “Herbier D’Antoine Laurent de Jussieu” at P, and according to Art. 9.1 of the ICN, we recognize it as the holotype of *R.roseus*. This change immediately supersedes (Art. 9.19) the previous typification by Romoleroux (1996). We treated further specimens of *R.roseus* at P as possible original material.

We found original material of *R.santarosensis* at NY, similar to the case of *R.holtenii* (see notes on *R.nubigenus*). According to Art. 9.3, 9.11 and 9.12, we select collection NY-429655 as the lectotype of *R.santarosensis* because it has the locality information corresponding to the protologue. The other specimen is treated here as possible original material of the name.

**Taxonomic notes.** In addition to the previous synonyms for this name (Romoleroux 1996; Romoleroux et al. 2014), we also consider R.nubigenusvar.subinermis, *R.rosiflorus*, and *R.santarosensis* as synonyms of *R.roseus*. The types of all these taxa have glabrous branches, stipules, petioles and leaves, broadly ovate to auriculate, reflexed stipules, trifoliolate leaves, ovate sepals, with long-acuminate apex, mostly bi- or trifurcate, and fruits with numerous, small drupelets like the type of *R.roseus*.

**Possible original material of *Rubusroseus* Poir.: Peru.***J. Dombey s.n.* (P-00162129 [image!]).

**Possible original material of *Rubussantarosensis* Kuntze: Bolivia.** 3000 m, 01–04 Apr 1892 (fr), *O. Kuntze s.n.* (NY-429654 [image!]).

**Specimens examined. Venezuela. • Táchira**: Cabeceras del Río Quinimarí, entre el pie del peñasco de la Peña de Pata de Judío, 2500–2800 m, 12 Jan 1968 (fl), *J.A. Steyermark, G.C.K. Dunsterville & E. Dunsterville 100819* (US-03733585, US-03733586). **Colombia. • Cauca**: Cordillera Central, Páramo de Juntas, 3300 m, 13 Oct 1961 (fl, fr), *J. Cuatrecasas & L. Willard 26437* (COL-000197689). **Ecuador. • Napo**: Parque Nacional Llanganates, vía Salcedo-Tena, 00°59.450'S, 78°17.950'W, 3233 m, 22 Feb 2015 (fl, fr), *Á.J. Pérez, N. Zapata & W. Santillán 8247* (QCA-234720). **Peru. • Piura**: Huancabamba, Carmen de la Frontera, quebrada Rosarios, 04°59.153'S, 79°22.815'W, 2300–2600 m, 22 May 2003 (fl, fr), *S.M. Baldeón-Malpartida & F. Neyra-Jiménez 5406* (USM-273409). **Bolivia. • La Paz**: Murillo, valle de Río Zongo, 16°08.000'S, 68°07.000'W, 2750 m, 08 Jan 1988 (fl, fr), *F. Grifo & J. Solomon 642* (QCA-92257). • **Cochabamba**: Ayopaya, Silapata, 2700 m, Dec 1935 (fr), *M. Cárdenas 3377* (US-00641907).

**Distribution.***Rubusroseus* is reported from the Andes of Venezuela, Colombia, Ecuador, Peru and Bolivia.

### ﻿42. *Rubusruizii* Focke, Abh. Naturwiss. Vereins Bremen 4: 162. 1874.

≡ Rubusnubigenusvar.ruizii (Focke) Focke, Biblioth. Bot. 72: 37. 1910.

= *Rubusweberbaueri* Focke, Biblioth. Bot. 72: 38. 1910, syn. nov. • Type. Peru. Huánuco: Huamalíes, “Berge südwestlich von Monzon” [mountains south-west of Monzon], 3200–3300 m, 12 Jun 1903 (fl, fr), *A. Weberbauer 3362* (holotype: B-10-0248171 [image!]).

**Type. Peru.***H. Ruiz 19/65* (neotype, designated here: MA-01-00812077!).

**Nomenclature notes.** The holotype of *R.ruizii* at B was destroyed in WWII, but we found two specimens at MA, previously annotated by F. Bolle as R.nubigenusvar.ruizii. The photograph of the holotype of *R.ruizii* at F allowed us to confirm the identity of the two specimens at MA as *R.ruizii*. We select specimen *Ruiz 19/65* as the neotype of *R.ruizii* according to Art. 9.8 of the ICN.

**Taxonomic notes.** Although Focke (1874) described *R.ruizii*, he later considered it as a variety of *R.nubigenus* (Focke 1910). However, considering the neotype, it differs from *R.nubigenus* by: grayish-tomentose branches with abundant, short prickles; narrow leaves and leaflets; whitish-tomentose leaf abaxial surface; and unarmed sepals. These are characters of *R.weberbaueri* Focke, and it is clear that they refer to the same species. As *R.ruizii* is the oldest legitimate name available, it should be used for the taxon (Art. 11).

**Photograph of original material of *Rubusruizii* Focke: Peru.** Pillao, *H. Ruiz 206* (F [image!]).

**Specimens examined. Peru. • San Martín**: Mariscal Cáceres, Distrito Huicungo, valle de Allpamachay, Cueva del Oso en la intersección de Vacas Blancas y Allpamachay, 07°58.628'S, 77°21.323'W, 3518 m, 25 Jun 2010 (fl), *B. León 5609* (USM-243308). • **Unknown**: Tambo Real, *H. Ruiz 19/64* (MA-01-00812078). **Bolivia. • La Paz**: Nor Yungas, Unduavi, 3300 m, Nov 1910 (fl, fr), *O. Buchtien 2858* (US-00641913). Yungas, 6000 ft, 1885 (fr), *H.H. Rusby 471* (NY-429650).

**Distribution.***Rubusruizii* is distributed in the Andes of southern Peru and Bolivia.

### ﻿43. *Rubusrusbyi* Britton, Bull. Torrey Bot. Club 17(1): 10. 1890.

**Type. Bolivia. La Paz**: Unduavi, 10000 ft., Oct 1885 (fl), *H.H. Rusby 2508* (holotype: NY-429653 [image!]).

**Specimens examined. Peru. • Pasco**: Oxapampa, Huancabamba, Parque Nacional Yanachaga-Chemillen, sector San Daniel, 10°25.767'S, 75°26.100'W, 3250–3450 m, 01 Mar 2008 (fl), *R. Vásquez, A. Monteagudo, A. Peña & J. Mateo 33855* (USM-235919). • **Cusco**: Paucartambo, Tres Cruces, Parque Nacional Manu, 3600–3700 m, 06 Mar 1991 (fl, fr), *A. Cano 4589* (USM-243308).

**Distribution.***Rubusrusbyi* is known from the Andes of southern Peru and Bolivia. We report here the presence of *R.rusbyi* for the first time in Peru.

### ﻿44. *Rubusschottii* Pohl ex Focke, Abh. Naturwiss. Vereins Bremen 4: 157. 1874.

**Type. Brazil.***Schott 5885* (lectotype, designated here: W-279047 [image!]; isolectotype: W-279046 [image!], W-279048 [image!]).

**Nomenclature notes.**Focke (1874) cited *Schott 5885* from two herbaria, thus a lectotype is required (Art. 9.3 of the ICN). Therefore, according to Art. 9.3, 9.11 and 9.12, we designate the collection W-279047 as the lectotype of *Rubusschottii* because it has fruits and shows the abaxial surface of a mature leaf.

**Specimens examined. Brazil. • Minas Gerais**: Caparaó, 24 Oct 1989 (fl), *R. Simao-Bianchini, J.R. Pirani, R. Mello-Silva & J.B. Fernandes 237* (SPF-68142). • **Espírito Santo**: Conceição do Castelo, Alto Bananal, 120 m, 22 Aug 1987 (fl), *G. Hatschbach & A.C. Cervi 51315* (MBM-120266).

**Distribution.***Rubusschottii* is reported from southern Brazil.

### ﻿45. *Rubussellowii* Cham. & Schltdl., Linnaea 2(1): 15. 1827.

**Type. Brazil.** Brasilia meridionalis, *Sellow s.n.* (lectotype, designated here: B-10-0248177 [image!]; isolectotype: HAL-98247 [image!]).

**Nomenclature notes.***Rubussellowii* requires a lectotype (Art. 9.3 of the ICN) because Schlechtendal and Chamisso (1827) cited two Sellow collections, from “Brasilia meridionalis” and “Montevideo” respectively. We located two specimens from “Brasilia meridionalis” in B and HAL. According to Art. 9.3, 9.11 and 9.12, we designate the specimen at B as the lectotype of *R.sellowii*, since it has the 5-foliolate leaf mentioned in the protologue.

**Specimens examined. Argentina. • Misiones**: Posadas, Nov 1907 (fl), *E.L. Ekman 1867* (US-03733713). **Paraguay. • [Guairá**]: Villarrica, *P. Jörgensen 3860* (US-03733711). **Brazil. • Santa Catarina**: Itapiranga, 4 km W of Popí, 200–350 m, 24 Feb 1957 (fl, fr), *L.B. Smith, R. Klein & J. Schnorrenberger 11766* (US-01351366).

**Distribution.***Rubussellowii* is reported in Argentina, Paraguay and southern Brazil.

### ﻿46. *Rubusurticifolius* Poir., Encycl. [J. Lamarck & al.] 6(1): 246. 1804 (as “*urticaefolius*”).

≡ *Dyctispermaurticifolius* (Poir.) Raf., Sylva Tellur.: 160. 1838. ≡ “Rubusurticifoliusvar.typicus Focke”, Biblioth. Bot. 18, Heft 83: 56. 1914, nom. inval.

= *Rubustrichomallus* Schltdl., Linnaea 13(2): 268. 1839. • Type. Mexico. Hacienda de la Laguna, Aug 1829 (fr), *C.J.W. Schiede s.n.* (lectotype, designated here: HAL-60486 [image!]).

= *Rubushassleri* Chodat, Bull. Herb. Boissier 7, App. 1: 66. 1899. ≡ Rubusurticifoliusvar.hassleri (Chodat) Focke, Biblioth. Bot. 18, Heft 83: 55. 1914. • Type. Paraguay. Feb 1885–1895 (fl, fr), *E. Hassler 1901* (lectotype, designated here: G-00640010 [image!] two sheets, isolectotypes: BM-000548793 [image!], P-682374 [image!]).

**Type. Peru.***J. Dombey s.n.* (holotype: P-00678396 [image!]).

**Nomenclature notes.** The situation of the typification of *R.urticifolius* is similar to that of *R.coriaceus* and *R.coriaceus*, where Poiret (1804) cited a collection from the Jussieu Herbarium. Therefore, according to Art. 9.1 of the ICN, we recognize the only collection of *R.urticifolius* with a label from the “Herbier D’Antoine Laurent de Jussieu” at P as the holotype of *R.urticifolius*. We treat further specimens from P as possible original material, and provide information on the typification of previous synonyms of this name.

The original material of *R.trichomallus* is at HAL (Braun and Wittig 2003), with two sheets annotated with this name. We select HAL-60486 as the lectotype of *R.trichomallus* (Art. 9.3, 9.11 and 9.12 of the ICN), while the other specimen is treated here as original material. Chodat (1899) did not cite any specimen in the protologue, but we traced back *Hassler 1901* which corresponds to the original description. We therefore designate the specimen at G as the lectotype of *R.hasslerii* (Art. 9.3, 9.11 and 9.12).

**Taxonomic notes.** The synonymy follows the revision of Fuks (1984).

**Original material of *Rubusurticifolius* Poir.: Peru.***J. Dombey s.n.* (P-00162125 [image!]); Lima, *J. Dombey s.n.* (P-00162123 [image!], P-00162124 [image!], P-00162126 [image!]).

**Original material of *Rubustrichomallus* Scthltdl.: Mexico.** Hacienda de la Laguna, Aug 1829 (fr), *C.J.W. Schiede s.n.* (HAL-107627 [image!]).

**Specimens examined. Venezuela. • Bolívar**: Río Anawaray-parú, vecindades del km 134 y campamento 134 al sur de El Dorado, 1300–1350 m, 25 Dec 1970 (fl, fr), *J.A. Steyermark, G.C.K. Dunsterville & E. Dunsterville* (US-03733792). **Colombia. • Valle del Cauca**: Río Digua Valley, between La Elsa and Río Blanco, 900 m, 2–5 Apr 1939 (fl, fr), *E.P. Killip* (US-03733754). **Ecuador. • Pichincha**: Quito, Nono, vía Nono-Tandayapa entre km 117–118, 00°01.967'S, 78°38.491'W, 1925 m, 26 Jul 2021 (fl, fr), *D. Espinel-Ortiz & H.G. Abad 279* (QCA-244015, QCA-7010801, QCA-7010802). **Peru. • Cajamarca**: San Ignacio, Huarango, El Convento, 05°13.000'S, 78°40.000'W, 1200–1600 m, 01 Jul 1996 (fl, fr), *J. Campos & E. Rodríguez 2836* (HUT-45736, USM-143879). **Argentina. • Misiones**: Posadas, “La Granja”, 14 Jan 1908, *E.L. Ekman 1873* (US-03733815). **Paraguay. • Itapúa**: Opposite Puerto Piray, 200 m, 23 Oct 1978 (fl, fr), *S.A. Renvoize 3215* (P-03340708, US-03733811). **Brazil. • Bahía**: Piatã, proximidades do riacho Toborou, 13°09.550'S, 41°45.917'W, 1060 m, 04 Nov 1996 (fl, fr), *D.J.N. Hind, H.P. Bautista, M.M. da Silva & L.P. de Queiroz 4042* (US-01351270).

**Distribution.***Rubusurticifolius* ranges from northern Central America across most of South America, from 200–2500 m asl.

### ﻿Excluded names

#### ﻿a. Rubusadenomallusvar.larecajanus Focke, Biblioth. Bot. 18, Heft 83: 52. 1914.

**Type. Bolivia. [La-Paz**]: Larecaja “viciniis Sorata, inter Laripata et monticulus Pancuasi” [near Sorata, between Laripata and small mountain Pancuasi], 2700–3100 m, Aug (fl, fr) 1859, *G. Mandon 658* (lectotype, designated here: K-000424915 [image!], isolectotype: BR-33828565 [image!]).

**Notes.**Focke (1914) cited two gatherings in the protologue: *Mandon 657* and *Mandon 658*. We found these specimens at K and BR. However, only *Mandon 658* matches the description based on the glands in the branches, while *Mandon 657* corresponds to *R.megalococcus*. Therefore, we designate *Mandon 658* at K as the lectotype of this name (Art. 9.3, 9.11 and 9.12 of the ICN).

It is possible that R.adenomallusvar.larecajanus is related to *R.megalococcus*, *R.bogotensis* or *R.adenothallus*. *Mandon 658* has abundant glands on the branches and leaves, which is a character of *R.bogotensis* and *R.adenothallus*. However, the shape and size of the leaves are similar to those of *R.adenothallus* and *R.megalococcus*, but the fruits with few, big drupelets are a character of *R.megalococcus* and *R.bogotensis*. Further studies are therefore required to clarify the identity of R.adenomallusvar.larecajanus.

#### ﻿b. *Rubusaenigmaticus* Focke, Meded. Rijks-Herb. 19: 55. 1913.

**Type. Bolivia.** No collection was cited by Focke (Herzog 1913).

**Notes.** Focke (Herzog 1913) described this taxon as a possible hybrid of *R.brierus* and *R.buchtienii* [= *R.megalococcus*]. He did not cite any specific collection, just that it was collected by Buchtien. We could not locate any specimen annotated by Focke, thus its identity is currently unclear.

#### ﻿c. *Rubuseriocarpus* Liebm., Vidensk. Meddel. Naturhist. Foren. Kjøbenhavn 1852: 162. 1853.

≡ Rubusoccidentalissubsp.eriocarpus (Liebm.) Focke, Abh. Naturwiss. Vereins Bremen 4: 147. 1875.

**Type. Mexico. Puebla**: Chinautla, 7000 ft, Jun (fl), *Liebmann s.n.* (syntype); Vulcanen Orizaba, 10000 ft, Sep (fl), *Liebmann s.n.* (syntype). **Oaxaca**: Cerro de Sempoaltepec, 8000–10000 ft, Jun (fr), *Liebmann s.n.* (syntype). **Unknown**: Jalapa, May, *Schiede s.n.* (syntype); Jalapa, Sep, *Lerma s.n.* (syntype); Mineral del Monte, *Ehrenberg s.n.* (syntype).

**Notes.**Tropicos.org (2024) treated *R.glaucus* as a synonym of *R.eriocarpus*, but we omitted the latter from the checklist as *R.glaucus* and *R.eriocarpus* may be different species (Carter et al. 2019; Huang et al. 2023; IPNI 2024; POWO 2024). The aim of this checklist was the South American species of *Rubus*, but *R.eriocarpus* was described from Mexico. Whether or not they are synonyms, the accepted name of the species in South America is *R.glaucus*. If *R.glaucus* and *R.eriocarpus* are synonyms, the name *R.glaucus* has priority over *R.eriocarpus* (Art. 11 of the ICN).

#### ﻿d. Rubusloxensisf.parvifolius Kuntze, Meth. Sp.-Beschr. Rubus: 117. 1879.

**Type.** Neu Granada, *J. Goudot s.n.* (holotype).

**Notes.**Kuntze (1879) cited a specimen collected by Goudot, previously identified as *R.loxensis*, at P. However, we were unable to locate any specimen annotated by Kuntze, or with this locality and identification. Based on the description, this name could be a synonym of *R.nubigenus* or *R.coriaceus*. The name needs to be typified to clarify its identity.

#### ﻿e. *Rubusporphyromallos* Focke, Repert. Spec. Nov. Regni Veg. 9: 235. 1911.

**Type.** “Andibus partis borealis Americaaustralis”. No collection was cited by Focke (1911a).

**Notes.**Focke (1911a, 1911b) cited no collections for this name, just mentioned that it is similar to *R.bogotensis* and occurs in the North Andes of South America. We could not locate any specimens annotated by Focke, thus the identity of *R.porphyromallos* is currently unclear. It may refer to a specimen similar or related to *R.pendulus* (Espinel-Ortiz et al. 2023).

#### ﻿f. Rubusschottiivar.pohlianus Focke, Abh. Naturwiss. Vereins Bremen 4: 158. 1874.

**Type. Brazil. Matto Grosso**: “In silva Matto grosso”, Cap Goyaz, *J.P.E. Pohl 1093* (holotype: W-279045 [image!]).

**Notes.**Focke (1874) cited *Pohl 1093* at W in the protologue, thus according to Art. 9.1 of the ICN, we recognize this specimen as the holotype of R.schottiivar.pohlianus. We require further studies to clarify the identity of this name.

#### ﻿g. Rubusurticifoliusvar.rosiflorus Vargas, Revista Univ. (Cuzco) 32(84): 266. 1943 (as “rosaeflorus”).

**Type. Peru. Cusco**: Convención, valle de Lucumayo, Amaibamba, 1900 m, 27 Jul 1943, *C. Vargas 3433* (hototype: CUZ-5585!; isotype: CUZ-5589!).

**Notes.** We require further studies to clarify the identity of this name.

## ﻿Conclusions

In this checklist, we recognized 46 species of *Rubus* from 110 names based on South American specimens published since 1767. Approximately 90% were published before 1990, when no holotype was required for a valid publication. As a result, 51 names required typification. Adams (Cafferty and Jarvis 2002), Fuks (1984) and Romoleroux (1996) typified 22 names, and we designated 22 lectotypes, 4 neotypes and 1 epitype. In addition, after careful examination, we proposed new synonyms explaining the reasoning behind each one and reported *R.azuayensis*, *R.laegaardii* and *R.rusbyi* for the first time from Peru. Some major changes introduced here were the restoration of *R.organensis* as a distinct species, the new name *R.neobrasiliensis* that replaced *R.brasiliensis* and the typification of *R.mollifrons* and *R.ruizii*. All in one, this annotated list will be the basis for future studies of *Rubus* in South America, especially monographic and evolutionary approaches, as well as catalogs or other disciplines that want to study *Rubus*.

## ﻿Additional information

### Author ORCIDs

David A. Espinel Ortiz https://orcid.org/0000-0003-3405-0375

Katya Romoleroux https://orcid.org/0000-0002-0679-9218

Tim Böhnert https://orcid.org/0000-0003-1415-7896

Maximilian Weigend https://orcid.org/0000-0003-0813-6650

### Data availability

All of the data that support the findings of this study are available in the main text or Supplementary Information.

## Supplementary materials

Supplementary material 1 Summary of accepted species of Rubus from South America, available names and their types Data type xlsx Explanation note Excel with three sheets summarizing the available names of *Rubus* from South America, their types and the valid species presented in the checklist. Each sheet is named after the information it contains. For example, the sheet "types" contains the hyperlinks to the respective online herbarium samples that are treated as types. https://binary.pensoft.net/file/1150701This dataset is made available under the Open Database License (http://opendatacommons.org/licenses/odbl/1.0/). The Open Database License (ODbL) is a license agreement intended to allow users to freely share, modify, and use this Dataset while maintaining this same freedom for others, provided that the original source and author(s) are credited.David A. Espinel Ortiz, Katya Romoleroux, Tim Böhnert, Maximilian Weigend
